# Handling Metadata in a Neurophysiology Laboratory

**DOI:** 10.3389/fninf.2016.00026

**Published:** 2016-07-19

**Authors:** Lyuba Zehl, Florent Jaillet, Adrian Stoewer, Jan Grewe, Andrey Sobolev, Thomas Wachtler, Thomas G. Brochier, Alexa Riehle, Michael Denker, Sonja Grün

**Affiliations:** ^1^Institute of Neuroscience and Medicine (INM-6), Institute for Advanced Simulation (IAS-6), JARA BRAIN Institute I, Jülich Research CentreJülich, Germany; ^2^Laboratoire d'informatique Fondamentale, UMR 7279, Centre National de la Recherche Scientifique, Aix-Marseille UniversitéMarseille, France; ^3^Institut de Neurosciences de la Timone, UMR 7289, Centre National de la Recherche Scientifique, Aix-Marseille UniversitéMarseille, France; ^4^Department of Biology II, Ludwig-Maximilians-Universität MünchenMartinsried, Germany; ^5^Institut for Neurobiology, Abteilung Neuroethologie, Eberhard-Karls-Universität TübingenTübingen, Germany; ^6^Institute of Neuroscience and Medicine (INM-6), Jülich Research CentreJülich, Germany; ^7^Theoretical Systems Neurobiology, RWTH Aachen UniversityAachen, Germany

**Keywords:** metadata management, reproducibility, analysis workflow, electrophysiology, data sharing, odML

## Abstract

To date, non-reproducibility of neurophysiological research is a matter of intense discussion in the scientific community. A crucial component to enhance reproducibility is to comprehensively collect and store metadata, that is, all information about the experiment, the data, and the applied preprocessing steps on the data, such that they can be accessed and shared in a consistent and simple manner. However, the complexity of experiments, the highly specialized analysis workflows and a lack of knowledge on how to make use of supporting software tools often overburden researchers to perform such a detailed documentation. For this reason, the collected metadata are often incomplete, incomprehensible for outsiders or ambiguous. Based on our research experience in dealing with diverse datasets, we here provide conceptual and technical guidance to overcome the challenges associated with the collection, organization, and storage of metadata in a neurophysiology laboratory. Through the concrete example of managing the metadata of a complex experiment that yields multi-channel recordings from monkeys performing a behavioral motor task, we practically demonstrate the implementation of these approaches and solutions with the intention that they may be generalized to other projects. Moreover, we detail five use cases that demonstrate the resulting benefits of constructing a well-organized metadata collection when processing or analyzing the recorded data, in particular when these are shared between laboratories in a modern scientific collaboration. Finally, we suggest an adaptable workflow to accumulate, structure and store metadata from different sources using, by way of example, the odML metadata framework.

## 1. Introduction

Technological advances in neuroscience during the last decades have led to methods that nowadays enable to simultaneously record the activity from tens to hundreds of neurons simultaneously, *in vitro* or *in vivo*, using a variety of techniques (Nicolelis and Ribeiro, 2002; Verkhratsky et al., 2006; Obien et al., 2014) in combination with sophisticated stimulation methods, such as optogenetics (Deisseroth and Schnitzer, 2013; Miyamoto and Murayama, 2015). In addition, recordings can be performed in parallel from multiple brain areas, together with behavioral measures such as eye or limb movements (Maldonado et al., 2008; Vargas-Irwin et al., 2010). Such recordings enable to study network interactions and cross-area coupling and to relate neuronal processing to the behavioral performance of the subject (Berenyi et al., 2013; Lewis et al., 2015; Lisman, 2015). These approaches lead to increasingly complex experimental designs that are difficult to parameterize, e.g., due to multidimensional characterization of natural stimuli (Geisler, 2008) or high dimensional movement parameters for almost freely behaving subjects (Schwarz et al., 2014). It is a serious challenge for researchers to keep track of the overwhelming amount of metadata generated at each experimental step and to precisely extract all the information relevant for data analysis and interpretation of results. Various aspects such as the parametrization of the experimental task, filter settings and sampling rates of the setup, the quality of the recorded data, broken electrodes, preprocessing steps (e.g., spike sorting) or the condition of the subject need to be considered. Nevertheless, the organization of these metadata is of utmost importance for conducting research in a reproducible manner, i.e., the ability to faithfully reproduce the experimental procedures and subsequent analysis steps (Laine, 2007; Peng, 2011; Tomasello and Call, 2011). Moreover, detailed knowledge of the complete recording and analysis processes is crucial for the correct interpretation of results, and is a minimal requirement to enable researchers to verify published results and build their own research on the previous findings.

To achieve reproducibility, experimenters have typically developed their own lab procedures and practices for performing experiments and their documentation. Within the lab, crucial information about the experiment is often transmitted by personal communication, through handwritten laboratory notebooks or implicitly by trained experimental procedures. However, at latest when it comes to data sharing across labs, essential information is often missed in the exchange (Hines et al., 2014; Open Science Collaboration, 2015). Moreover, if collaborating groups have different scientific backgrounds, for example experimenters and theoreticians, implicit domain-specific knowledge is often not communicated or is communicated in an ambiguous fashion that leads to misunderstandings. To avoid such scenarios, the general principle should be to keep as much information about an experiment as possible from the beginning on, even if information seems to be trivial or irrelevant at the time. Furthermore, one should annotate the data with these metadata in a clear and concise fashion.

In order to provide metadata in an organized, easily accessible, but also machine-readable way, Grewe et al. (2011) introduced odML (open metadata Markup Language) as a simple file format in analogy to SBML in systems biology (Hucka et al., 2003), or NeuroML in neuroscientific simulation studies (Gleeson et al., 2010; Crook et al., 2012). However, lacking to date is a detailed investigation on how to incorporate metadata management in the daily lab routine in terms of (i) organizing the metadata in a comprehensive collection, (ii) practically gathering and entering the metadata, and (iii) profiting from the resulting comprehensive metadata collection in the process of analyzing the data. Here, we address these points, both, conceptually, and practically in the context of a complex behavioral experiment that involves neuronal recordings from a large number of electrodes that yield massively parallel spike and local field potential (LFP) data (Riehle et al., 2013). To illustrate how to organize a comprehensive metadata collection (i), we introduce in Section 2 the concept of metadata, and demonstrate the rich diversity of metadata that arise in the context of the example experiment. To demonstrate why the effort of creating a comprehensive metadata collection is time well spent, we describe in Section 3 five use cases that summarize where the access to metadata becomes relevant when working with the data (iii). Section 4 provides detailed guidelines and assistance on how to create, structure and hierarchically organize comprehensive metadata collections (i and ii). Complementing these guidelines, we provide a thorough practical introduction on how to embed a metadata management tool, such as the odML library, into the experimental and analysis workflow in the Supplementary Material. Finally, in Section 5 we critically contrast the importance of proper metadata handling against its difficulties, and derive future challenges.

## 2. Organizing metadata in neurophysiology

Metadata are generally defined as data describing data (Baca, 2008; Merriam-Webster, 2016). More specifically, metadata are information that describe the conditions under which a certain dataset has been recorded (Grewe et al., 2011). Ideally all metadata would be available machine-readable at a single location that is linked to the corresponding recorded dataset. The fact that such central, comprehensive metadata collections are not common practice already today is by no means a sign of negligence on the part of the scientists, but is explained by the fact that in the absence of conceptual guidelines and software support, such a situation is extremely difficult to achieve given the high complexity of the task. Already the fact that an electrophysiological setup is composed of several hardware and software components, often from different vendors, imposes the need to handle multiple files of different formats. Some files may even contain metadata that are not machine-readable and -interpretable. Furthermore, performing an experiment requires the full attention of the experimenters which limits the amount of metadata that can be manually captured online. Metadata that arise unexpectedly during an experiment, e.g., the cause of a sudden noise artifact, are commonly documented as handwritten notes in the laboratory notebook. In fact, handwritten notes are often unavoidable, because legal regulations of some countries, e.g., France^1^, require the documentation of experiments in the form of a handwritten laboratory notebook.

**Figure 1:**
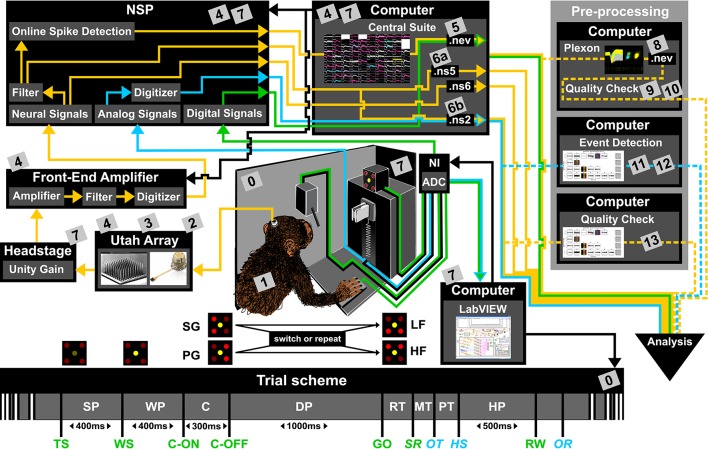
**Overview of the reach-to-grasp setup**. Bottom: Time line of the experiment indicating the sequence of presented stimuli (WS, C-ON, C-OFF, GO, RW), the behavioral epochs (SP, WP, C, DP, RT, MT, PT, MP) and registered behavioral events (TS, SR, OT, HS). Fixed durations of behavioral epochs are specified in the time line. The abbreviations used in the trial scheme are explained in Table 1. The sketch of the experimental setup above illustrates the involved hard and software components (boxes) as well as the signal flow. The signal flow is composed of two streams of signals: Recording and processing of the neuronal signals (yellow arrows) and the signals related behavior (green and blue arrows). The controller of each component is indicated by black arrows. The gray boxes indicate files that contain metadata generated or updated at this stage (cf. numbers in boxes to labels in Table 2 for details). The signal flow of the neuronal data starts at the level of the Utah array, continues with the headstage, the Front-End Amplifier, the Neural Signal Processor (NSP), and ends with saving the data as three Blackrock specific data file formats (.nev, .ns2 and .ns5/.ns6) using Central Suite running on the data acquisition PC. The stream of behavioral signals is split into two parallel pathways. One of them contains the analog signals (force and displacement sensors), colored in blue, and the other one the digital signals (LEDs, table switch, and reward control), colored in green. The correct sequence of trial events (presentation of stimuli), the setting of the load force of the object and the performed behavior (timing, movement) are monitored (blue and green arrows) and controlled (black arrows) online via LabVIEW on a second setup control PC. The digitization of the signals is performed via an Analog-Digital converter (ADC) of National Instruments (NI) which is upstream to the setup control PC. The ADC is also upstream to NSP which is used to save all digital or digitized signals into the nev data file via Central Suite. In parallel the analog signals of the load and displacement sensors of the object are fed directly into the NSP and saved into the .ns2 file. Consecutive preprocessing of the neuronal as well as the behavioral signals (flow of preprocessed signals marked by dashed lines) is performed offline on separate PCs, such as the spike sorting with the Plexon offline Spike Sorter or other preprocessing steps with custom programs (e.g., “Quality Check” or “Event Detection”). Image of Utah array courtesy of Blackrock Microsystems.

To present the concept of metadata management in a practical context, we introduce in the following a selected electrophysiological experiment. For clarity, a graphical summary of the behavioral task and the recording setup is provided in Figure 1, and a list of task-related abbreviations (e.g., behavioral conditions and events) is listed in Table 1. First described in Riehle et al. (2013) and Milekovic et al. (2015), the experiment employs high density multi-electrode recordings to study the modulation of spiking and LFP activities in the motor/pre-motor cortex of monkeys performing an instructed delayed reach-to-grasp task. In total, three monkeys (*Macaca mulatta*; 2 females, L, T; 1 male, N) were trained to grasp an object using one of two different grip types (side grip, SG, or precision grip, PG) and to pull it against one of two possible loads requiring either a high (HF) or low (LF) pulling force (cf. Figure 1). In each trial, instructions for the requested behavior were provided to the monkeys through two consecutive visual cues (C and GO) which were separated by a one second delay and generated by the illumination of specific combinations of 5 LEDs positioned above the object (cf. Figure 1). The complexity of this study makes it particularly suitable to expose the difficulty of collecting, organizing and storing metadata. Moreover, metadata were first organized according to the laboratory internal procedures and practices for documentation, while the organization of the metadata into a comprehensive machine-readable format was done a-posteriori, after the experiment was completed. To work with the data and metadata of this experiment one has to handle on average 300 recording sessions per monkey, with data distributed over 3 files per session, and metadata distributed over 5 files per implant, at least 10 files per recording and one file for general information of the experiment (cf. Table 2). This situation imposed an additional complexity to reorganize the various metadata sources.

**Table 1:** **Trial scheme parameters of the reach-to-grasp experiment**.

**Abbreviation**	**Written-out**	**Definition**
**REQUESTED BEHAVIOR (GRIP AND FORCE TYPES)**		
SG	Side grip	Indicated by the illumination of the two right outer red LEDs
PG	Precision grip	Indicated by the illumination of the two left outer red LEDs
LF	Low force	Pull force needed for low object load, indicated by the illumination of the two bottom outer red LEDs
HF	High force	Pull force needed for high object load, indicated by the illumination of the two upper outer red LEDs
**TRIAL EVENTS**		
TS	Trial start	Time point where monkey self-initiates a trial by pressing a table switch
WS	Warning signal	Time point where the central yellow LED of the visual cue system is illuminated to focus the monkey's attention toward the upcoming cues
C-ON	Cue-on	Time point of first cue where two of four red outer LEDs of the visual cue system are illuminated indicating the grip or force type
C-OFF	Cue-off	Time point where first cue is turned off
GO	Go signal	Time point of second cue where two of four red outer LEDs of the visual cue system are illuminated indicating the missing behavioral type
SR	Switch release	Time point where table switch is released by the monkey indicating movement onset
OT	Object touch	Time point where object is touched by the monkey measured by the force and displacement sensors of the object
HS	Hold start	Time point where monkey reached holding position of the object
RW	Reward	Time point where monkey receives the trial reward
OR	Object release	Time point where monkey releases the object and returns its hand to the table switch for the next TS
**TRIAL PERIODS**		
SP	Start period	Period between TS and WS (400 ms)
WP	Waiting period	Period between WS and C-ON (400 ms)
C	Cue period	Period between C-ON and C-OFF (300 ms)
DP	Delay period	Period between C-OFF and GO (1000 ms)
RT	Reaction time	Period between GO and SR
MT	Movement time	Period between SR and OT
PT	Pull time	Period between OT and HS
HP	Hold period	Period between HS and RW (500 ms)

*The list is organized in trial scheme parameters defining the requested behavior, trial scheme parameters defining the trial events, and trial scheme parameters defining the trial periods. The list provides the abbreviations for each trial scheme parameter used in Figure 1 and a short definition of the parameter*.

**Table 2:** **Metadata sources of the reach-to-gasp experiment**.

**Label**	**File**	**Format**	**Software**	**Generation**	**Content**	**# Files**
**METADATA FILES WHICH ARE GENERATED BEFORE THE RECORDING PERIOD STARTS**						
0	Project specific info file	xls	Excel	Manually	General information on the experiment	1/exp.
1	Subject/array specific info file	xls	Excel	Manually	Information on the monkey (profile, surgery, training)	1/monkey or 1/array
2	Electrode configuration file	txt	Text editor	Manually	Information on the electrodes of the Utah array	1/array
3	Electrode configuration file	txt	Text editor	Manually	Information on the anatomical placement of the array	1/array
4	Blackrock configuration file	xls	Excel	Manually	Information on the Blackrock hard- and software properties	1/array
**METADATA FILES WHICH ARE GENERATED DURING THE RECORDING PERIOD**						
5	Neural event file	nev	Central suite	Automatically	Information on the Blackrock hard- and software settings (Incl. spike waveforms and event times)	1/rec.
6a	Neural signal file	ns5/6	Central suite	Automatically	Continuous neuronal signals in high resolution	1/rec.
6b	Neural signal file	ns2	Central suite	Automatically	Continuous LFP signals, and analog signals of the force and displacement sensors of the object	1/rec.
7	Recording specific file	xls	Excel	Manually	Information on the recording	1/rec.
**METADATA FILES WHICH ARE GENERATED AFTER THE RECORDING PERIOD (PREPROCESSING STEPS)**						
8	Neural event file	nev	Plexon	Semi-automatically	Information on the Blackrock hard- and software settings (incl. spike waveforms, event times, and spike sorting results)	1/rec.
9	Spike sorting results	txt	Text editor	Manually	Information on the spike sorting results	1/rec.
10	Spike sorting results	mat	MATLAB	Semi-automatically	Quality assessment of the spike sorting results (preprocessing step)	1/rec.
11	Behavioral event file	mat	MATLAB	Semi-automatically	Detection of OT, HS and OR	1/rec.
12	Behavioral event file	mat	MATLAB	Semi-automatically	Detection of object load force	1/rec.
13	Quality assessment file	hdf5	Python	Semi-automatically	Quality assessment of LFP signals for different frequency bands	(1/frq-band) per rec.

*The label of each source file is used in Figure 1 to indicate at which experimental stage metadata are generated or updated. Additionally, the table summarizes information about the software used to generate each source file, whether the generation process was automatized, the content of each source file, and how many files were generated for each source within the complete reach-to-grasp experiment*.

To give a more concrete impression of the painstaking detail that needs to be considered while planning and organizing a comprehensive metadata collection, we provide an exhaustive description of the experiment in the Supplementary Material. To illustrate the level of complexity of metadata management in this example, Figure 1 outlines the different components of the experimental setup, the signal flow, the task and the trial scheme. In addition, the heterogeneous pieces of metadata and the corresponding files that contain them in the absence of a comprehensive metadata collection are listed and described in Table 2. Here, all metadata source files are labeled by numbers and appear in Figure 1 wherever they were generated. If labels appear multiple times, they were iteratively enriched with information obtained from the corresponding components of the setup.

In such a complex experiment, the relevance of some metadata does not become immediately apparent and is sometimes underestimated. For example, the immediate relevance of each minute detail of the experimental setup are of little interest for the interpretation of a single recording, and only when one attempts to rebuild the experiment, these metadata become valuable. Apart from that, any piece of metadata can become highly relevant for screening and selecting datasets according to specific criteria at a later stage. Different experiments produce different sets of metadata and it is therefore not possible to provide a detailed list of metadata that needs to be collected from any given type of experiment. Nevertheless, the general principle should be to keep as much information about an experiment as possible from the beginning on, even if information seems to be trivial or irrelevant at the time. Based on our experience gathered from various collaborations, we compiled a list of guiding questions to help scientists defining their optimal collection of metadata for their use scenario, e.g., reproducing the experiment and/or the analysis of the data, or sharing the data:

- What metadata would be required to replicate the experiment elsewhere?
- Is the experiment sufficiently explained, such that someone else could continue the work?
- If the recordings exhibit spurious data, is the signal flow completely reconstructable to find the cause?
- Are metadata provided which may explain variability (e.g., between subjects or recordings) in the recorded data?
- Are metadata provided which enable access to subsets of data to address specific scientific questions?
- Is the recorded data described in sufficient detail, such that an external collaborator could understand them?

For the example experiment presented in Figure 1, we used these guiding questions to control if the content of the various metadata sources was sufficient, and enriched these where necessary. The process of planning the relevant metadata content and to organize the metadata into a comprehensive collection can be time-consuming, especially if the process happened after the experiment was performed.

In the following, we will outline use cases that illustrate why the time for creating a comprehensive metadata collection is well spent, nevertheless.

## 3. Advantage of a comprehensive metadata collection: use cases

Based on the example experiment (Figure 1), we present five use cases that demonstrate the common scenarios in which access to metadata becomes important. For the implementation of each use case, we contrast the scenarios before and after having organizing metadata in a comprehensive collection:

- *Scenario 1*: Metadata are organized in different files and formats which are stored in a metadata source directory.
- *Scenario 2*: Metadata sources of scenario 1 are compiled into a comprehensive collection, stored in one file per recording using a standard file format.

We introduce the protagonists acting in the use cases and characterize their relationship:

- *Alice:* She is the experimenter who built up the setup, trained the monkeys and carried out the experimental study. She has programming experience in MATLAB and performs the preprocessing step of spike sorting.
- *Bob:* He is a data analyst and a new member of Alice's laboratory. He has programming experience in MATLAB and Python. His task is to support Alice in implementing the preprocessing and first analysis of the data.
- *Carol:* She is a theoretical neuroscientist and an analyst of an external group that collaborates with Alice's laboratory. She is an experienced Python programmer. Information exchange with Alice's group is limited to phone, email and video calls, and few in-person meetings. She is not an experimentalist and therefore not used to work with real neuronal data. She never participated in an experiment, and thus never experienced the workflow of a typical recording session.

Each of the use cases below illustrates a different aspect of the analysis workflow: enrichment of metadata information, metadata accessibility, selection and screening of datasets, and formal queries to reference metadata.

### 3.1. Use case 1: enrichment of the metadata collection

**Figure 2:**
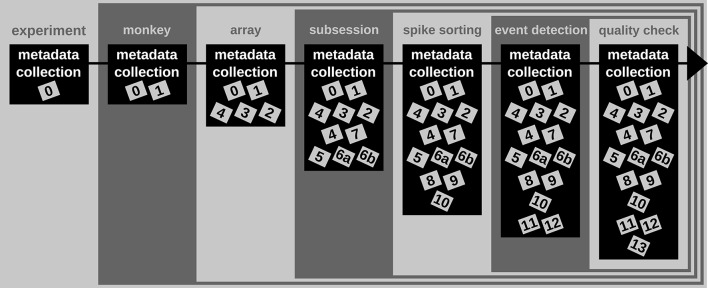
**Enrichment of the reach-to-grasp metadata collection over time**. Small gray and labeled boxes represent files which contain metadata (cf. Table 2). The metadata collection is first generated at the beginning of the experiment and contains only information constant for the complete experiment. With each monkey, the metadata collection is enriched by monkey-specific information that remains constant for the time of the experiment. With each array implantation, information about that array is included in the metadata collection. During or directly after the recording of each session the metadata collection is enriched by information specific to the recording. Several preprocessing steps are then performed on the recorded data (here spike sorting, event detection, and quality check). The newly generated metadata are integrated step-by-step into the metadata collection and enrich the information available for subsequent data analysis.

*A difficult aspect of metadata management is that one cannot know all metadata in advance that are necessary to ensure the reproducibility of the experiment and the data analysis. Thus, the metadata collection needs to be enriched when new and updated information becomes available (see Figure* *2**). An example for such an enrichment of a metadata collection is the integration of results and parameters of preprocessing steps applied to the recorded data, since these are usually performed long after the primary data acquisition (cf. Table* *2**). Use case 1 demonstrates the advantage of using a standard file format when a metadata collection is enriched with information from successive preprocessing steps*.

In one of the first meetings with Alice, Bob learned that a number of preprocessing steps are performed by Alice. One of these is to identify and extract time points of the behavioral events describing the object displacement in each trial (OT, HS, OR, see Table 1) from the continuous force and displacement signals which were saved in the ns2-datafile (label 6b in Figure 1 and Table 2). For this, the signals were loaded by a program Alice developed in MATLAB to perform this preprocessing step. The extracted behavioral events are saved for each recording session along with the processing parameters in a respective .mat file (labels 11 in Figure 1 and Table 2). It is important that these preprocessing results are readily accessible to all collaboration partners including Bob, because they are needed to correctly interpret the timeline and behavior in each trial.

In scenario 1, Alice does not create a comprehensive metadata collection, and Bob has to deal with the fact that the behavioral events are not only not stored along with the digitally available trial events (e.g., TS, see Table 1) in the recorded nev-file (label 5 in Figure 1 and Table 2), but also saved in the custom-made mat-file which requires him to write a corresponding loading routine. In scenario 2, Alice saved the digital, directly available trial events into one comprehensive metadata collection per recording. With the preprocessing of each recording, she enriched then the corresponding primary collection with all results and parameters of the behavioral trial event extraction. As a result, the access to all trial events becomes easier for Bob, so that he is able to quickly scan the behavior in each trial.

In summary, a comprehensive metadata collection has the advantage that it bundles metadata that conceptually belongs together, and simplifies access by combining metadata into one single, standard file format. Moreover, the flexible enrichment of a comprehensive metadata collection simplifies the organization of metadata that originate from multiple versions of performing a preprocessing step, e.g., as in the case of offline spike sorting (see Supplementary Material). With this, a comprehensive metadata collection not only simplifies the reproducibility of Alice's work (e.g., repetition of preprocessing steps), but also guarantees a better reproducibility for the collaboration with Bob (e.g., standardized access to parameters and results of preprocessing steps).

### 3.2. Use case 2: metadata accessibility

*Complex experimental studies usually include organizational structures on the level of the file system (e.g., directories) and file format to store all data and metadata. Even in cases where all data are very well organized, metadata are usually distributed over several files and formats (see Table* *2**). Use case 2 demonstrates how a standard file format of a comprehensive metadata collection improves the accessibility and readability of metadata*.

When Bob first arrived in the lab, Alice explained to him the structure of the data and metadata of the experimental study. Unfortunately, Bob could not start working on the data directly after the meeting with Alice. He made of course several notes during this meeting, but due to the complexity of the information he forgot where to find the individual pieces of metadata in sufficient detail.

In scenario 1, Bob starts to scan all different metadata sources to regain an overview of what can be found where. This means that he has not only to go through several files, but also that he needs to deal with several formats, and that he needs to understand the different design of each file. In scenario 2, Bob knows that all metadata are collected and stored in one file per recording which is readable using standard software, e.g., a text editor. To access again an overview of the available metadata, he can open a file of any recording, screen its content or search for specific metadata.

Thus, the metadata organization in scenario 1 has the following disadvantages: (i) it is difficult to keep track of which metadata source contains which piece of information; (ii) not every metadata source is readable with a simple, easy accessible software tool (e.g., the binary data files, .nev and .nsX; labels 5, 6a, and 6b in Figure 1 and Table 2, respectively); (iii) not every software tool offers the option to search for a specific metadata content. In contrast, in scenario 2, a comprehensive, standardized and searchable organization of the metadata guarantees a complete and easy access to all metadata related to an experiment even over a long period of time. This simplifies the long-term comprehensibility of Alice's experiment not only for herself, but also for all further group members and collaboration partners.

### 3.3. Use case 3: selection of datasets

*Data of an experiment can usually be used to address multiple scientific questions. In this context, it is necessary to select datasets according to certain criteria which are defined by the requirements and constraints of the scientific question. These criteria are usually represented by metadata related to one recording session and generated during or after the recording period (see Table* *2**). Use case 3 demonstrates how a comprehensive metadata collection with a standard file format supports the automatic selection of datasets according to defined selection criteria*.

Alice reported that she had the feeling that the weekend break influences the monkey's performance on Mondays and asked Bob to investigate her suspicion. To solve this new task, Bob first wants to identify the recording sessions for each weekday (criterion 1) which were recorded with standard task settings (criterion 2) and under the standard experimental paradigm (instructed delayed reach-to-grasp task with two cues; criterion 3). To automatize the data selection, Bob writes a Python program where he loops through all available recording sessions, uses criterion 1 to identify the weekday, and adds each session name to a corresponding list if in the session criteria 2 and 3 were fulfilled.

To write this program, Bob uses in scenario 1 the knowledge he gained from the manual inspection in use case 2 to identify for the chosen selection criteria the different metadata source files. Criterion 1 was stored in the .nev data file (label 5 in Figure 1 and Table 2) which Bob can access via the data loading routine. Criteria 2 and 3 are instead stored in the recording-specific spreadsheet (label 7 in Figure 1 and Table 2). To automatize their extraction, Bob additionally needs to write a spreadsheet loading routine, because there is no standard loading routine available for the homemade structure of the spreadsheet. In contrast, in scenario 2, Bob extracts all three criteria for each recording from one comprehensive metadata file using an available loading routine of the chosen standard file format.

In summary, compared to scenario 1, scenario 2 improved the workflow of selecting datasets according to certain criteria in two aspects: (i) to check for the selection criteria only one metadata file per recording needs to be loaded, and (ii) to extract metadata stored in a standardized format a loading routine is already available. Thus, in scenario 2 the scripts for automatized data selection are less complicated, which improved the reproducibility of the operation.

### 3.4. Use case 4: metadata screening

*Sometimes it can be helpful to gain an overview of the metadata of an entire experiment. Such a screening process is often negatively influenced by the following aspects. (i) Some metadata are stored along with the actual electrophysiological data. (ii) Metadata are often distributed over several files and formats. (iii) Some metadata need to be computed from other metadata. All three aspects slow down the screening procedure and complicate the corresponding code. Use case 4 demonstrates how a comprehensive metadata collection improves the speed and reproducibility of a metadata screening procedure*.

**Figure 3:**
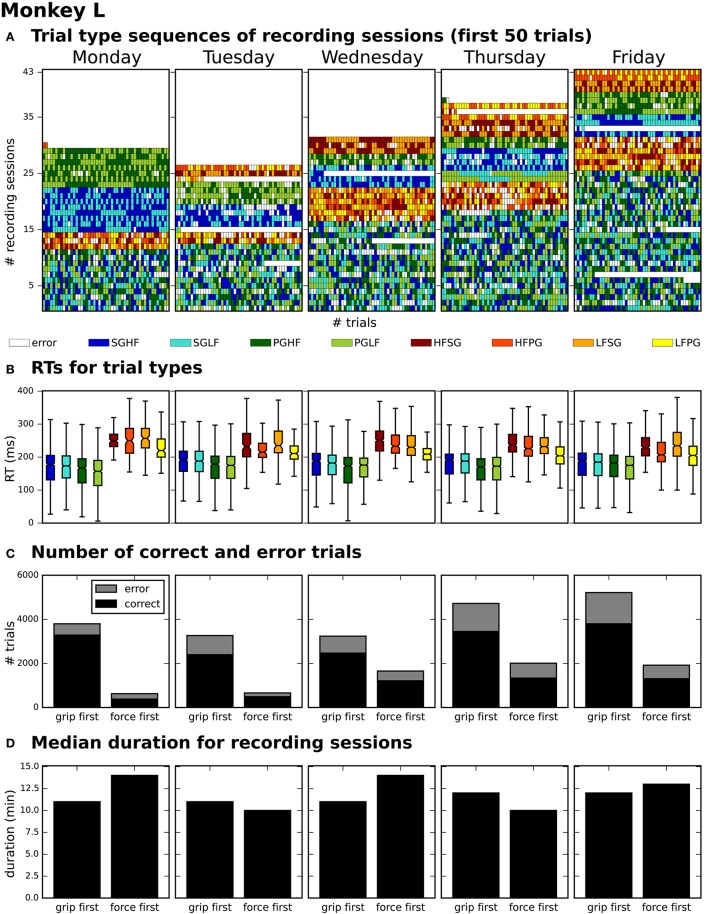
**Overview of reach-to-grasp metadata summarizing the performance of monkey L on each weekday**. **(A)** displays the order of trial sequences of the first 50 trials (small bars along the x-axis) for each of all sessions (rows along the y-axis) recorded on the corresponding weekday. Each small bar corresponds to a trial and its color to the requested trial type of the trial (see color legend below subplot **(A)**; for trial type acronyms see main text). **(B)** summarizes the median reaction times (RTs) for all trials of the same trial type (see color legend) of all sessions recorded on the corresponding weekday. **(C)** shows for each weekday the total number of trials of all sessions differentiating between correct and error trials (colored in black and gray) and between sessions where the monkey was first informed about the grip type (grip first) compared to sessions where the first cue informed about the force type (force first). **(D)** displays for each weekday the median recording duration of the grip first and force first sessions.

After generating for each weekday a corresponding list of recording file names (see use case 3, Section 3.3), Bob would like to generate an overview figure summarizing the set of metadata that best reflect the performance of the monkey during the selected recordings (Figure 3). This set includes the following metadata: the RTs of the trials for each recording (Figure 3B), the number of correct vs. error trials (Figure 3C), and the total duration of the recording (Figure 3D). To exclude a bias due to a variable distribution of the different task conditions, Bob also wants to include the trial type combinations and their sequential order (Figure 3A).

To create the overview figure in scenario 1, Bob has to load the .nev data file (label 5 in Figure 1 and Table 2) in which most selection criteria are stored, and additionally the .ns2 data file (label 6b in Figure 1 and Table 2) to extract the duration of the recording. For both files, Bob is able to use available loading routines, but for accessing the desired metadata he always has to load the complete data files. Depending on the data size this processing can be very time consuming. In scenario 2, Bob is able to directly efficiently extract all metadata from one comprehensive metadata file without having to load the neuronal data in parallel.

In summary, compared to scenario 1, the workflow of creating an overview of certain metadata of an entire experiment is improved in scenario 2 by reducing the number of metadata files which need to be screened to one per recoding, and by drastically lowering the run time to collect the criteria used in the figure. In addition, Bob benefits from better reproducibility as in use case 3 (Section 3.3).

### 3.5. Use case 5: metadata queries for data selection

*It is common that datasets are analyzed not only by members of the experimenter's lab, but also collaborators. Two difficulties may arise in this context. First, the partners will often base their work on different workflow strategies and software technologies, making it difficult to share their code, in particular code that is used to access data objects. Second, the geographical separation represents a communication barrier resulting from infrequent and impersonal communication by telephone, chat, or email, requiring extra care in conveying relevant information to the partner in a precise way. Use case 5 demonstrates how a standard format used to save the comprehensive metadata collection improves cross-lab collaborations by formalizing the communication process through the use of queries on the metadata*.

Alice has detected a systematic noise artifact in the LFP signals of some channels in recording sessions performed in July (possibly due to an additional air-conditioning). As a consequence, Alice decided to exclude recordings performed in July from her LFP spectral analyses. Alice collaborates with Carol to perform complementary spike correlation analyses on an identical subset of recordings in order to find out if the network correlation structure is affected by task performance. To ensure that they analyze exactly the same datasets, the best data selection solution is to rely on metadata information that is located in the data files, rather than error-prone measures such interpreting the file name or file creation date.

In scenario 1, since Alice and Carol use different programming languages (MATLAB and Python), they need to ensure that their routines extract the same recording date. This procedure will require to provide corresponding cross-validations between their MATLAB and Python routines to extract identical dates.

**Figure 4:**
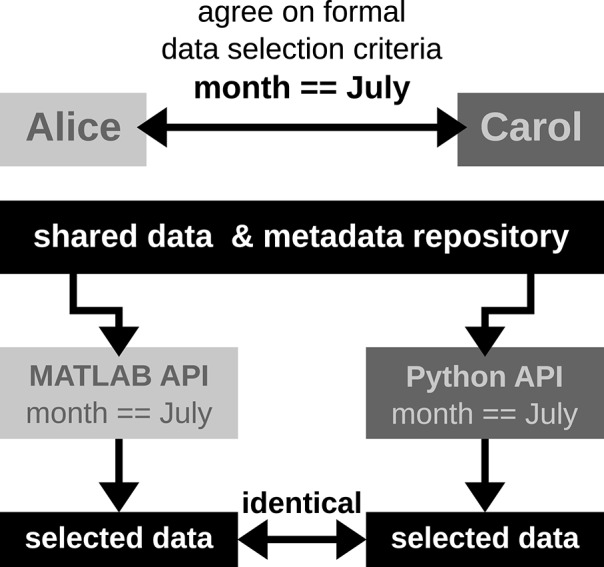
**Schematic workflow of selecting data across laboratories based on available API for a comprehensive metadata collection**. In this optimized workflow, Alice and Carol are both able to identically select data from the common repository by screening the comprehensive metadata collection for the selection criteria (month == July) via the available APIs for MATLAB and Python. Such a formalized query in data selection supports a well-defined collaborative workflow across laboratories.

In scenario 2, Alice and Carol agree on a concrete formal specification of the dataset selection (Figure 4) via the comprehensive metadata collection. In such a specification, metadata are stored in a defined format that reflects the structure of key-value pairs: in our example, Alice would specify the data selection by telling Carol to allow for only those recording sessions where the key Month has the exact value July. For the chosen standardized format of the collection, the metadata query can be handled by an application program interface (API) available both at the MATLAB and Python levels for Alice and Carol, respectively. Therefore, the query is guaranteed to produce the same result for both scientists. The formalization of such metadata queries will result in a more coherent and less error-prone synchronization between the work in the two laboratories.

## 4. Guidelines for creating a comprehensive metadata collection

The five use cases illustrate the importance and usefulness of a comprehensive metadata collection in a standardized format. One effort to develop such a standardized format is the odML project, which implements a metadata model proposed by Grewe et al. (2011). The odML project supports a software library (the odML library) for reading, writing, and handling odML files through an API with language support for Python, Java and MATLAB. The remainder of this paper complements the original technical publication of the software (Grewe et al., 2011) by illustrating in a tutorial like style its practical use in creating a comprehensive metadata collection. We demonstrate this process using the metadata of the described experiment as a practical example. We show how to generate a comprehensive metadata file and outline a workflow to enter and maintain metadata in a collection of such files. Although we are convinced that the odML library is particularly well designed to reach this goal, the concepts and guidelines are of general applicability and could be implemented with other suitable technology as well.

### 4.1. The odML metadata model

**Figure 5:**
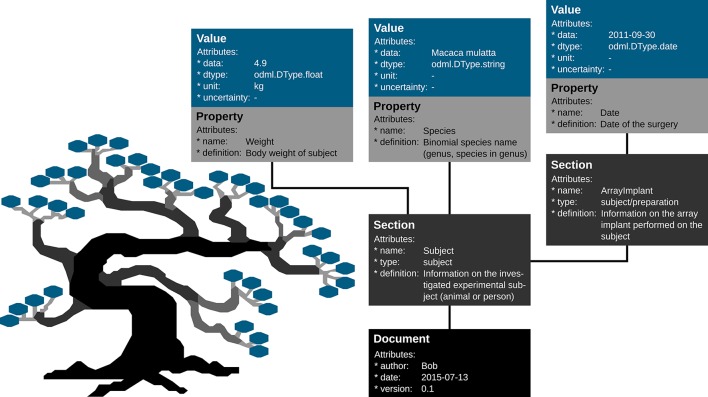
**Example of the structural design of an odML file**. The displayed Sections, Properties and Values are a subset of the odML Subject branch of the reach-to-grasp experiment (Subject_Demo.odml). Metadata are stored in the “data” attributes of odML Value objects, which also provide additional attributes to describe the datatype (dtype), the unit or the uncertainty of the Value. Which metadata are stored in the Values is defined by the connected Property object via its “name” and “definition” attribute. These Property-Value/s pairs that contain metadata of a similar context are grouped together into a Section object that is given a “name” and a “definition.” If a sub-context needs to be defined in more detail, Sections can also group other Sections together containing the corresponding metadata again in Property-Value/s pairs. To finalize the tree structure, Sections which are highest in the hierarchy are grouped together into a Document object which can state additional information on the odML file, such as who created it (“author”), when it was created (“date”), or which code or template version was used to create it.

Metadata can be of arbitrary type and describe various aspects of the recording and preprocessing steps of the experiment (cf. Figure 1 and Table 2). Nevertheless, all metadata can be represented by a key-value pair, where the key indicates the type of metadata, and the value contains the metadata or points to a file or a remote location where the metadata can be obtained (e.g., for image files). Consequently, the odML metadata model is built on the concepts of Properties (keys) paired with Values as the structural foundation of any metadata file. A Property may contain one or several Values. Its name is a short identifier by which the metadata can be addressed and its definition can be used to give a textual description of metadata stored in the Value(s). Properties that belong to the same context (e.g., description of an experimental subject) are grouped in so called Sections which are specified by their name, type and a definition. Sections can further be nested, i.e., contain sub-sections and thus form a tree-like structure. At the root of the tree is the Document which contains information about the author, the date of the file and a version. Figure 5 illustrates how a subset of the experimental metadata is organized in an odML file. The design concepts presented here can be transferred to many other software solutions for metadata handling. We chose the odML library because it is comparatively generic and flexible, which makes it well-suited for a broad variety of experimental scenarios, and which enabled us to introduce it as metadata framework in all our collaborations. For extensive details on the odML metadata model in addition to Grewe et al. (2011), we provide a tutorial of the odML API as part of the odML Python library^2^.

### 4.2. Metadata strategy: distribution of information

In preparing a metadata strategy for an experiment, one has to decide if and how information should be distributed over multiple files. For example, one could decide to either generate (i) several files for each recording, (ii) a single file per recording, or (iii) a single file that comprises a series of recordings. The appropriate approach depends on both, the complexity of metadata and the user's specific needs for accessing them. In the following, we will exemplify situations which could lead to one of the three different approaches:

- If the metadata of a preprocessing step are complex, combining them with the metadata related to the initial data acquisition could be confusing. One could instead generate separate files for the preprocessing and the recording. The downside of this approach is that the availability of both files needs to be assured.
- In an experiment where each single recording comprises a certain behavioral condition, the amount of metadata describing each condition is complex, and recordings are performed independently from each other, a single comprehensive metadata file per recording should be used. This approach was chosen for the example experiment described in Section 2.
- If one recording is strongly related to, or even directly influences, future recordings (e.g., learning of a certain behavior in several training sessions), then one single comprehensive metadata file should cover all the related recordings. Even metadata of preprocessing steps could be attached to this single file.

### 4.3. Metadata strategy: structuring information

Organizing metadata in a hierarchical structure facilitates navigation through possibly complex and extensive metadata files. The way to structure this hierarchy strongly depends on the experiment, the metadata content, and the individual demands resulting from how the metadata collection should be used. Nevertheless, there are some general guidelines to consider (based on Grewe et al., 2011):

- Keep the structure as flat as possible and as deep as necessary. Avoid Sections without any Properties.
- Try to keep the structure and content as generic as possible. This enables the reuse of parts of the structure for other recording situations or even different experiments. Design a common structure for the entire experiment, or even across related studies, so that the same set of metadata filters can be used as queries in upcoming analyses. If this is not possible and multiple structures are introduced, for instance, because of very different task designs, create a Property which you can use to determine which structure was used in a particular file (e.g., Property “UsedTaskDesign”: Value “TaskDesign_01”).
- Create a structure that categorizes metadata clearly into different branches. Make use of the general components of an electrophysiological experiment (e.g., subject, setup, hardware, software, etc.), but also classify metadata according to context or time (e.g., previous knowledge, pre- and postprocessing steps).
- In order to describe repeating entities like an experimental trial or an electrode description, it is advisable to separate constant properties from those that change individually. Generate one Section for constant features and unique Sections for each repetition for metadata that may change.

**Figure 6:**
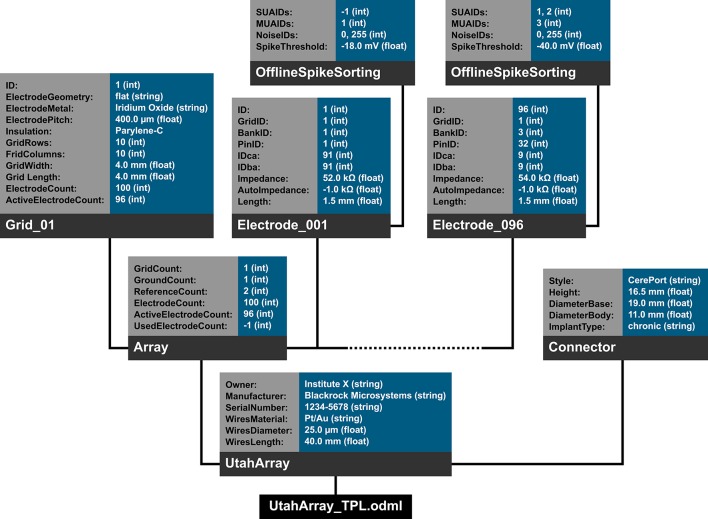
**Schematic view of the tree structure of the odML template for a Utah array**. Color code of boxes matches color code of odML objects in Figure 5. To simplify the schema, odML Properties and their values are listed in blocks for each odML section and additional attributes of the odML objects (e.g., definitions) are not displayed. Likewise, the remaining 94 odML Sections of the Utah Array electrodes (“Electrode_002” to “Electrode_095”) are left out and only indicated by the dashed line. Based on the size of the odML template for a Utah array, which is only one branch of a final odML file of the reach-to-grasp study, one can imagine the complexity of a complete reach-to-grasp odML file.

In the following we will illustrate these principles with a subset of the metadata related to our example experiment (Section 2). Using the nomenclature of the odML metadata framework, we structured the metadata of an Utah array into the hierarchy of Sections which is schematically displayed in Figure 6.

The top level is called “UtahArray.” A Utah array is a silicon-based multi-electrode array which is wired to a connector. General metadata of the Utah array, such as the serial number (cf. “SerialNo” in Figure 6) are directly attached as Properties to the main Section (guideline i).

More detailed descriptions are needed for the other components of the Utah array. The hierarchy is thus extended with a Section for the actual electrode array and a Section for the connector component. For both Utah array components, there are different fabrication types available which differ only slightly in their metadata configuration. For this reason, we named the Sections generically “Array” and “Connector” (guideline ii) and specified their actual fabrication type via their attached Properties (e.g., “Style”).

The fabrication type of the “Array” is defined by the number and configuration of electrodes. In our experiment a Utah array with 100 electrodes (96 connected, 4 inactive) arranged on a 10 × 10 grid, supplied with wires for two references and one ground was used. It is, however, possible to have a different total number of electrodes and even an array split into several grids with different electrode arrangements. Nevertheless, all electrodes or grids can be defined via a fixed set of properties that describe the individual setting of each electrode or grid. To keep the structure as generic as possible, we registered, besides the total number of electrodes references and grounds, the number of active electrodes, and the number of grids as Properties of the (level-2-) Section “Array” (guideline ii). Within the “Array” Section, (level-3-) Sections named “Electrode_XXX” for each electrode and grid are attached, each containing the same Properties, but with individual Values for that particular electrode. This design makes it possible to maintain the structure for other experiments where the number and arrangement of electrodes and grids might be different (guidelines ii and iv). The electrode IDs of a Utah array are numbered consecutively, independent of the number of grids. In order not to further increase the hierarchy depth, a Property “Grid_ID” in each “Electrode_XXX” Section identifies to which grid the electrode belongs to, instead of attaching the electrodes as (level-4-) Sections to its Grid and Array parent Sections (guideline i).

The example of the Utah array demonstrates the advantage of a meaningful naming scheme for Sections and Properties. To prevent ambiguity, any Section and Property name at the same level of the hierarchy must be unique. However, a given name can be reused at different hierarchy depths. This reuse can facilitate the readability of the structure and make its interpretation more intuitive. The “UtahArray” Section (Figure 6) demonstrates a situation in which ambiguous Section and Property names are useful, and where they must be avoided. The Sections for the individual electrodes of the array are all on the same hierarchy level. For this reason, their names need to be unique which is guaranteed by including the electrode ID into the Section name (e.g., “Electrode_001”). In contrast, the Property names of each individual electrode Section, such as “ID,” “GridID,” etc., can be reused. Similarly, the results of the offline spike-sorting can be stored in a (level-4-) Section “OfflineSpikeSorting” below each electrode Section. Using the identical name for this Section for each electrode is helpful, because its content identifies the same type of information. In the Supplementary Material, we show for the odML framework hands-on how one can make use of recurring Section or Property names to quickly extract metadata from large and complex hierarchies.

### 4.4. Metadata strategy: workflow

**Figure 7:**
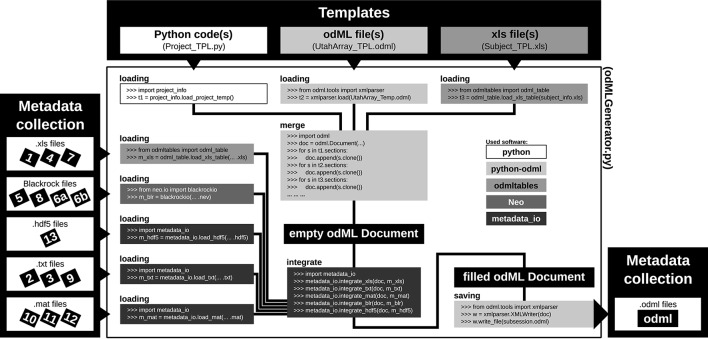
**Schematic workflow for generating odML files in the reach-to-grasp experiment**. The Templates box (top): illustrates three out of six template parts which are used to build the complete, but (mostly) empty odML Document combining metadata of one recording session in the experiment. The three possible template formats (script, odML file, and spreadsheet) are indicated within the gray shaded boxes (left to right, respectively). The Metadata collection box (left) illustrates all metadata sources of a recording session (black labeled small boxes) ordered according to file formats (white boxes). Labels of metadata sources are listed in Table 2. The central large white box shows the workflow of the odML generator routine (odMLGenerator.py). Code snippets used at the different steps of this workflow are illustrated in the smaller gray scaled boxes whose colors represent the software used for the code (see legend right of center). The black colored boxes indicate files or file stages. The workflow consists of 5 steps: (i) loading all template parts, (ii) merging all template parts into one empty odML Document, (iii) loading all metadata sources of a session, partially with custom-made routines (metadata_io), (iv) integrating all metadata sources into the empty odML Document using custom-made routines (metadata_io), and (v) saving the filled odML Document as odML file of the corresponding recording session. The Metadata collection box (right) illustrates the reduction of all metadata sources of one recording session to one metadata source (odML file).

For most experiments it is unavoidable that metadata are distributed across various files and formats (Figure 1 and Table 2). To combine them into one or multiple file(s) of one standard format, one not only needs to generate a meaningful structure for organizing the metadata of the experiment, but also to write routines that load and integrate metadata into the corresponding file(s). For reasons of clarity and comprehensibility we argue to separate these processes. Figure 7 illustrates and summarizes the corresponding workflow for the example experiment using the odML library.

**Figure 8:**
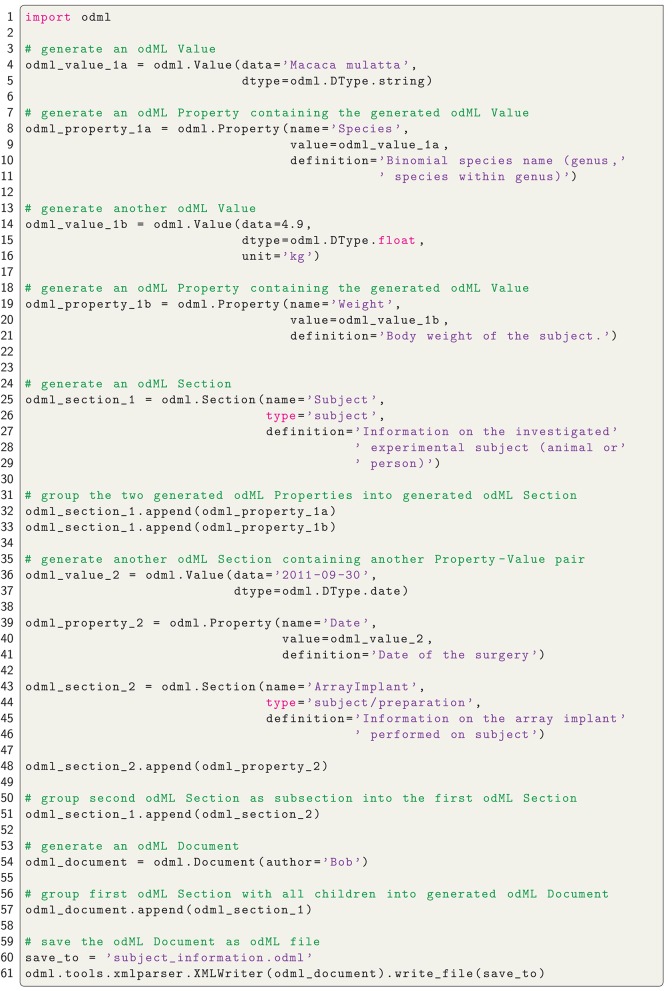
**Python code to create an odML file**. Python code to create the Subject_Demo.odml which is schematically shown in Figure 5.

**Figure 9:**
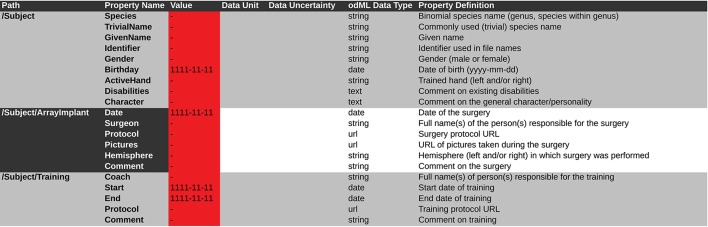
**Screen shot of a spreadsheet that can be automatically transferred to a corresponding odML template**. This example is generated from the template of the Subject branch (Subject_TPL). The .xls file representation is used to fill the template part with the corresponding metadata of the reach-to-grasp experiment (compare to Figure 5). Default values which should be manually changed to actual metadata are marked in red. To be able to directly use such a .xls file as template and later directly translate it to the odML format one should at least include the Section Definition as additional column [e.g., for “Path: /Subject” add “Section Definition: Information on the investigated experimental subject (animal or person)”].

As a first step, it is best to write a template in order to develop and maintain the structure of the files of a comprehensive metadata collection. In the odML metadata model, a template is defined as an “empty” odML file in which the user determined the structure and attributes of Sections and Properties to organize the metadata, but filled it with dummy Values. To create an odML template one can use (i) the odML Editor, a graphical user interface that is part of the odML Python library (see supplement), (ii) a custom-written program based on the odML library (Figure 8), or (iii) a spread-sheet software (see Figure 9), e.g., Excel (Microsoft Corporation). Especially for large and complex structures, (ii) or (iii) are the more flexible approaches to generate a template. For (iii) we developed the odML-tables package^3^ which provides a framework to convert between odML and spreadsheets saved in the Excel or CSV format. To simplify editing templates it is advisable to create multiple smaller templates (e.g., into the top-level Sections). These parts can then be handled independently and are later easily merged back into the final structure. This approach facilitates the development and editing even of large odML structures. The top of Figure 7 illustrates how three templates of the example experiment (Project_Temp, UtahArray_Temp, and Subject_Temp) representing the three possible template formats (i-iii) are merged into one large “empty” odML file via the Python odML library.

Once a template is created, as a second step, the user has to write or reuse a set of routines which compile metadata from the various sources and integrate them into a copy of the template and save them as files of the comprehensive metadata collection (see Figure 7, left part). Although, it would be desirable if the complete workflow of loading and integrating metadata into one or multiple file(s) of a comprehensive metadata collection would be automatized, in most experiments it cannot be avoided that some metadata need to be entered by hand (see Section 2). Importantly, we here avoid direct manual modification of the comprehensive metadata files. Instead, the final metadata collection is always created from a separate file containing manually entered metadata. This approach has the advantage that, if at one point the structure needs to be changed, these manual entries will remain intact. Practically, manual metadata entries are collected into a source file that is in a machine-readable format, such as text files, CSV format, Excel, HDF5^4^, JSON^5^, or directly in the format of the chosen metadata management software (e.g., odML), and use the corresponding libraries for file access to load and integrate them into the file(s) of the comprehensive metadata collection. In the example experiment we stored manual metadata in spreadsheets that are accessible by the odML-tables package. In the special case that these manual metadata entries are constant across the collection (e.g., project information, cf. Table 2), one can even reduce the number of metadata sources by entering the corresponding metadata directly into the templates (e.g., Project_Temp in Figure 7). This avoids unnecessary clutter in the later compilation of metadata and facilitates consistency across odML files. Metadata that are not constant across odML files should be preferably stored in source files that adhere to standard file formats which can be loaded via generic routines. For this reason, all spreadsheet source files in the presented experiment were designed to be compatible with the odML-tables package (see Subject_Temp in Figure 7 and as example in Figure 9). Furthermore, the files generated by the Blackrock data acquisition system as well as the results of the LFP quality assessment are loadable via the file interfaces of the Neo Python library (Garcia et al., 2014) which provides standardized access to electrophysiological data. Nevertheless, for the example experiment it was still necessary to write also custom loading and integration routines from scratch to cover all metadata from the various sources (e.g., .hdf5 and .mat files for results of the various preprocessing steps in Figure 7).

In summary, this two stage workflow of first generating templates, and then filling them from multiple source files guarantees flexibility and consistency of the metadata collection over time. In particular, in a situation where the structure needs to be changed at a later time (e.g., if new metadata sources need to be integrated) one only needs to adapt or extend the template as well as the code that fills the template with metadata in order to generate a consistent, updated metadata collection from scratch. If the metadata management can be planned in advance, one should attempt to optimize the corresponding workflow in the following aspects:

- Use existing templates (e.g., the Utah array odML template) to increase consistency with other experimental studies.
- Keep the number of metadata sources at a minimum.
- Avoid hidden knowledge in the form of handwritten notes or implicit knowledge of the experimenter by transferring such information into a machine-readable format (e.g., standardized Excel sheets compatible with odML-tables) early on.
- Automatize the saving of metadata as much as possible.

## 5. Discussion

We have outlined how to structure, collect and distribute metadata of electrophysiological experiments. In particular, we demonstrated the importance of comprehensible metadata collections (i) to facilitate enrichment of data with additional information, including post-processing steps, (ii) to gain accessibility to the metadata by pooling information from various sources, (iii) to allow for a simple and well-defined selection of data based on metadata information using standard query mechanisms, (iv) to create textual and graphical representations of sets of related metadata in a fast manner in order to screen data across the experiment, and (v) to formalize communication in collaborations by means of metadata queries. We illustrated how to practically create a metadata collection from data using the odML framework as example, and how to utilize existing metadata collections in the context of the five use cases.

**Figure 10:**
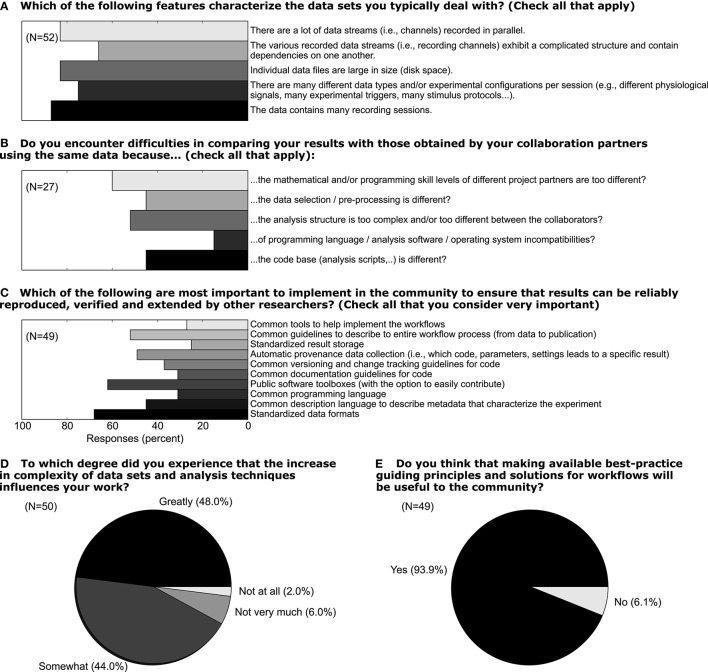
**Responses to five selected questions taken from a survey among scientists who deal with electrophysiological data sets**. The online survey was hosted on “Google Drive” and a total of 52 responders filled the questionnaire in the time period June 12 through October 17, 2011 (Associate/full professor: 18%, Assistant professor: 22%, Post-doc: 28%, PhD student 30%, no response 2%; self-reported). For questions A–C multiple answers could be given by a single person. The number of responders *N* is provided separately for each question.

In light of the increasing volume of data generated in complex experiments, neuroscientists, in particular in the field of electrophysiology, are facing the need to improve the workflows of the daily scientific life (Denker and Grün, in press). In this context, the aspects of handling metadata for electrophysiological experiments described above should be considered as part of such workflows. We conducted a survey among members of the electrophysiology and modeling community (*N* = 52) in 2011 to better understand how scientists think about the current status of their workflow, which aspects of their work could be improved, and to what extent these researchers would embrace efforts to improve workflows. In Figure 10 we show a selection of survey responses that are closely related to the role of metadata in setting up such workflows. 48% of responders reported that the increased complexity of data sets greatly influences their work (question D). When asked about which features characterize their data, it is obvious that multiple factors of complexity come into play, including the number of sessions, data size, dependencies between different data records (question A). In total, 93% believed that making available best-practice guidelines and workflow solutions would be beneficial for the community (question E). Handling metadata in the odML framework represents one option in designing best-practice for building such a workflow (question C). In fact, 46% of responders believed that a common description of metadata would be required to achieve that scientific work can be reproduced, verified and extended by other researchers. At present, 44% of researchers stated also that they find it difficult to compare their results to those obtained by other researchers working on the same or similar data due to differences in preprocessing steps and data selection (question B; cf., use case 5). The results of the full survey can be found under http://www.csn.fz-juelich.de/survey.

While the storage of metadata to improve the overall experimental and analysis workflow is technically feasible, it cannot solve the intrinsic problem of identifying the metadata in an experiment, collecting the individual metadata, and pooling them in an automatic way. All of these steps are not trivial and are time-consuming by nature, in particular when organizing metadata for a particular experiment for the first time. Currently, no funding is granted for such tasks, and software tools that support the automated generation and usage of metadata are largely missing. Therefore, rightly the question may arise whether it is truly worth the effort. Through the illustration of use cases, we hope to have convinced the reader that indeed numerous advantages are associated with a well-maintained metadata collection that accompanies the data. For the individual researcher these can possibly be best summarized by the ease of organizing, searching and selecting datasets based on the metadata information. Even more advantages are gained for collaborative work. First, an easily accessible central source for metadata information ensures that all researchers access the exact same information. This is particularly important if metadata are difficult to access from the source files, hand-written notebooks or via a specialized program code, or if the metadata need to be collected from different locations. Second, the communication between collaborators becomes more precise by the strict use of defined property-value pairs, lowering the probability for unintended confusion. Last but not least, once the metadata collection structure, content and method of creation are defined by the experimenter, metadata entry and compilation will in general run very smoothly, be less error prone, and will even save time in comparison to traditional methods. Thus, we believe that there are a number of significant advantages that justify the initial investment of including automated metadata handling as part of the workflow of an electrophysiological experiment. The survey results presented above reveal that these advantages are also increasingly recognized by the community. Furthermore, as scientists we have the obligation to properly document our scientific work in a clear fashion that enables the highest degree of reproducibility. Reproducibility becomes increasingly recognized by publishers and funding agencies (Morrison, 2014; Candela et al., 2015; Open Science Collaboration, 2015; Pulverer, 2015), such that appropriate resources of time and man-power allocated to produce more sophisticated data management will become a necessity. It is thus not only important to gain experience in how to document data properly, but also to produce better tools that reduce the time investment required for these steps.

As a result of our experience with complex analysis of experiments in systems neuroscience as reported in this paper, one of our recommendations is to record as much information about the experiment as possible. This may seem in contrast to efforts specifying *minimal information* guidelines in the life sciences (MIBBI; Taylor et al., 2008, MINI; Gibson et al., 2009). However, those initiatives target the use case where data are uploaded to a public database, and their goal is to achieve a balance of information detail such that the minimally sufficient information is provided to make the data potentially useful for the community. They are not meant as guidelines for procedures in the laboratory. To ensure reproducibility of the primary analysis, but also in the interest of future re-use of the data, we argue that it is highly desirable to store all potentially relevant information about an experiment.

We have exemplified the practical issues of metadata management using the odML metadata framework as one particular method. Similar results could be obtained using other formats. RDF^6^ is a powerful standard approach specifically designed for semantic annotation of data. Many libraries and tools are available for this format, and in combination with ontologies it is highly suitable for standardization. However, efficiently utilizing this format requires elaborate technology that is not easy to use. Simpler formats like JSON^7^ or YAML^8^ are in many respects similar to the XML schema used for odML, and we would expect that they have been used in individual labs to realize approaches similar to the one presented here. We are, however, not aware of specific tools available for the collection of metadata that use these formats. In addition, none of these alternative formats provide specific support for storing measured quantities as odML does. Using a combination of XML and HDF5^9^ has been proposed to define a format for scientific data (Millard et al., 2011), which could in principle be used for metadata collection. However, requiring extensive schema definitions it is much less flexible and lightweight than odML. Solutions for the management of scientific workflows, like Taverna^10^, Kepler^11^, VisTrails^12^, KNIME^13^, Wings^14^ (Badia et al., 2015), are targeted toward standardized and reproducible data processing workflows. We are focusing here on the management of experimental metadata, and a consideration of data processing would go beyond this scope. Workflow management systems could be utilized for managing metadata, but their suitability for the collection of metadata during the experiment seems limited. Nevertheless, the approach we have described here must ultimately be combined with such systems, as the development of automated workflows depends critically on the availability of a metadata collection. Likewise, our approach is suitable for combination with provenance tracking solutions like Sumatra (Davison et al., 2014).

Indeed, while we believe that using a metadata framework, such as odML, represents an important step toward better data and metadata management, it is also clear that this approach has a number of potential improvements. A natural step would be for odML to become an intrinsic file format for the commercially available data acquisition systems, such that their metadata are instantly available for inclusion in the user's hierarchical tree. In this context, the odML format so far has been adopted in the connectome file format^15^, the Relacs data acquisition and stimulation software^16^, and the EEGBase database for EEG/ERP data^17^.

Likewise, the availability of interfaces for easy odML export in popular experiment control suites, vendor-specific hardware or generic software, such as LabVIEW, would greatly speed up the metadata generation. In addition, a repository for popular terminologies and hardware devices has been initiated by the German Neuroinformatics Node^18^, which is open for any extensions by the community. More importantly, in conditions where the details of recording or post-processing steps may change over time, it is essential to keep track also of the versions of the code that generated a certain post-processing result, including the corresponding libraries used by the code and installed on the computer executing the code. Popular solutions to version control (e.g., git) or provenance tracking (e.g., Sumatra; Davison et al., 2014) offer mechanisms to keep track of this information. However, it is up to the user to make sure that information about the correct version numbers or hash values provided by these systems are saved to the file containing the metadata collection to guarantee that metadata can be linked to its provenance trail. The more direct integration of support for metadata recording into the various tools performing the post-processing would allow to automatize this process, leading to a more robust metadata collection, paired with enhanced usability.

A perhaps more challenging problem is devising mechanisms that link the metadata to the actual data objects they refer or relate to. This issue becomes particularly clear in the context of the Neo library (Garcia et al., 2014), which is an open-source Python package that provides data objects for storing electrophysiological data, along with file input/output for common file formats. The Neo library could be used to read a particular spike train that relates to a certain unit ID in the recording, while the corresponding odML file contains for each unit ID the information about the assigned unit type (SUA, MUA, or noise) and the signal-to-noise ratio (SNR) obtained by the spike sorting preprocessing step. A common task would now be to link these two pieces of information in a generic way. Currently, it is up to the user to manually extract the SNR of each neuron ID from the metadata, and then annotate the spike train data with this particular piece of metadata. This is a procedure that is time-consuming, and again may be performed differently by partners in a collaboration causing incoherence in the workflow. Recently, the NWB format (Teeters et al., 2015) was proposed as a file format to store electrophysiological data with a detailed, use-case specific data and metadata organization. In contrast, the NIX file format^19^ (Stoewer et al., 2014) was proposed as a more general solution to link data and metadata already on the file level. In this approach metadata are organized hierarchically as in odML, but can be linked to the respective data stored in the same file. This enables relating data and metadata meaningfully to facilitate and automate data retrieval and analysis.

Finally, convenient manual metadata entry is an important requirement for collecting metadata of an experiment. In the case of odML, while the existing editor enables researchers to fill in odML files, a number of convenience features would not only reduce the amount of time required to collect the information, but could also provide further incentives to store metadata in the odML format. An example for the former could be an editor support for templates such that new files with default values may be created quickly, while an example for the latter could be to provide more flexible ways to display metadata in the editor. The odML-tables library, which can transform the hierarchical structure of an odML file to an editable flat table in the Excel or CSV format, is one current attempt to solve these issues in the odML framework. The conversion of odML to commonly known spreadsheets increases the accessibility of odML for collaborators with little programming knowledge. Another particularly notable project aimed to improve the manual entries of metadata during an experiment is the odML mobile app (Le Franc et al., 2014) that runs on mobile devices that are easy to carry around in a lab situation.

The odML framework by itself is of a general nature and can easily be used in other domains of neuroscience than electrophysiology, or even other fields of science. An obvious use case would be to store metadata of neuroscientific simulation experiments. As we witness a similar increase in the complexity of *in silico* data, providing adequate metadata records to describe these data gains importance. However, datasets emerging from simulations differ in the composition of their metadata in terms of the more advanced technical descriptions required to capture the mathematical details of the employed models (e.g., descriptions using NeuroML; Gleeson et al., 2010; Crook et al., 2012), and by the fact that simulations are often described on a procedural rather than a declarative level (e.g., descriptions based on PyNN; Davison et al., 2008). How these can be best linked to more generic standards for metadata capture and representation, and what level of description of metadata is adequate in this scenario, remains a matter of investigation supported by use-cases, as performed here for experimental data. A common storage mechanism for metadata of experimental and simulated data would simplify their comparison, a task that is bound to become increasingly important for the future of Computational Neuroscience. Using a well-defined, machine-readable format for metadata brings the potential for integration of the information across heterogeneous datasets, for example in larger databases or data repositories.

In summary, the complexity of current electrophysiological experiments forces the scientific community to reorganize their workflow of data handling, including metadata management, to ensure reproducibility in research (Stodden et al., 2014). Readily available tools to support metadata management, such as odML, are a vital component in constructing such workflows. It is our responsibility to propagate and incorporate these tools into our daily routines in order to improve workflows through the principle of co-design between scientists and software engineers.

## Author contributions

LZ, MD, and SG designed the research. LZ, TB, and AR performed the research. LZ, MD, JG, FJ, ASo, ASt, and TW contributed to unpublished software tools. All authors participated in writing the paper.

### Conflict of interest statement

The authors declare that the research was conducted in the absence of any commercial or financial relationships that could be construed as a potential conflict of interest.

## Footnotes

^1^http://www.cnrs.fr/infoslabos/cahier-laboratoire/docs/cahierlabo.pdf

^2^https://github.com/G-Node/python-odml

^3^https://github.com/INM-6/python-odmltables

^4^https://www.hdfgroup.org

^5^http://www.json.org

^6^https://www.w3.org/RDF/

^7^http://www.json.org/

^8^http://yaml.org/

^9^https://www.hdfgroup.org/HDF5/

^10^http://www.taverna.org.uk/

^11^https://kepler-project.org/

^12^http://www.vistrails.org/

^13^https://www.knime.org/knime/

^14^http://www.wings-workflows.org/

^15^https://www.nitrc.org/projects/cff/

^16^http://www.relacs.net/

^17^https://eegdatabase.kiv.zcu.cz/

^18^http://portal.g-node.org/odml/terminologies/v1.0/terminologies.xml

^19^http://www.g-node.org/nix
