# Supplementary material for: Handling Metadata in a Neurophysiology Laboratory
Source: Front Neuroinform. 2016 Jul 19;10:26. doi: 10.3389/fninf.2016.00026 (PMC4949266; doi:10.3389/fninf.2016.00026)
Supplement: Supplementary file 1 [file Presentation1.pdf]

frontiers

Supplementary Material: Handling Metadata in a Neurophysiology Laboratory

Lyuba Zehl *, Florent Jaillet, Adrian Stoewer, Jan Grewe, Andrey Sobolev, Thomas Wachtler, Thomas Brochier, Alexa Riehle, Michael Denker, and Sonja Grün

*Correspondence:
Lyuba Zehl
l.zehl@fz-juelich.de

1 A COMPLEX NEUROPHYSIOLOGICAL EXPERIMENT

To analyze electrophysiological data and to relate the neuronal data to behavior the full details of the experiment, the experimental setup including the detailed signal flows need to be known. In the main text, we decided to put only a comprised description together with a figure of the setup Figure 1 and two complementary tables (Table 1 and Table 2). For the sake of completeness, we here give a more detailed description of the example experiment. The information given is organized according to the different phases of such an experiment and their relevance in respect to the metadata use cases outlined in the main text.

1.1 The task

Three monkeys (*Macaca mulatta*; 2 females, L, T; 1 male, N) were trained to grasp an object using one of two different grip types (side grip, SG, or precision grip, PG) and to pull it against one of two possible loads requiring either a high (HF) or low (LF) pulling force. In each trial, instructions for the requested behavior were provided to the monkeys through two consecutive visual cues (C and GO) which were separated by a one second delay and generated by the illumination of specific combinations of 5 LEDs positioned above the object. Information about the design and the mechanical engineering of the apparatus (e.g. the system providing the visual cue) were collected into a project specific info spreadsheet by the experimenter (label 0 in Figure 1, and Table 2). The experimental trial scheme including all behavioral events, behavioral periods, and stimuli are illustrated at the bottom of Figure 1 and described in Table 1. The corresponding metadata, such as the timing of the trial events, the typical duration of each period as well as their definitions and convenient abbreviations, were also collected in the project specific info spreadsheet.

1.2 The pre-recording period

When the monkey was fully trained in the task, a 100-electrode Utah array (Blackrock Microsystems, Salt Lake City, UT, USA) was surgically implanted in the motor cortex contralateral to the working hand. Details on the array (e.g. serial number, geometry, insulation, connector type) were collected in a Blackrock configuration spreadsheet by the experimenter (label 4 in Figure 1, and Table 2). Information about each electrode (e.g. ID, spatial location, impedance) was provided by the supplier (Blackrock Microsystems) in a non-machine-readable format, and therefore was transferred into an electrode configuration text file (label

2 in Figure 1 and Table 2). To be able to compare recordings across monkeys, a generalized order of the electrode IDs with respect to the individual anatomical placement of the array on the cortical surface was made by the experimenter and saved in a second array-specific text file (label 3 in Figure 1 and Table 2). All information about the training (e.g., duration, trainer, approach) and the surgery (e.g., pre-medication, surgeon, anesthesia) was collected in handwritten protocols. Later, key information about surgery and training (e.g., training duration, date of the surgery, implanted hemisphere) was extracted from these protocols and transferred, along with the links to the original files, into the subject or array specific info spreadsheet (label 1 in Figure 1 and Table 2). The subject or array specific info spreadsheet also included profile information for each monkey (e.g. birthday, species, name, working hand).

1.3 The recording period

The recording period lasted for at least half a year for each monkey. Recording sessions were performed on a daily basis, 5 days per week, and each lasted for about 2 hours. Within each recording day, data were recorded in 4 to 8 sessions, each saved in a set of 3 data files (.nev, .ns5/.ns6, and .ns2; labels 5, 6a, and 6b in Figure 1, and Table 2). Each session had a recording duration of about 15 min and was composed of 100 to 200 trials of a specified task condition. A task condition is defined by the order of the cue presentations (grip-cue first or force-cue first), the combination of 1, 2 or 4 trial types (PG/HF, PG/LF, SG/HF, SG/LF, HF/PG, HF/SG, LF/PG, LF/SG) and their sequence of presentation in the session (random or block design). The abbreviations and the respective numerical codes for the trial types and task conditions were again collected in the project specific info spreadsheet (label 0 in Figure 1, and Table 2).

The task condition was selected for each session by the experimenter depending on the mood and motivation of the monkey and the scientific question to be addressed. During some recording days, additional complementary experiments were performed, such as mapping the receptive fields (by passively moving (parts of) the limb or by tactile stimulation, see Riehle et al. 2013), or performing intra-cortical micro-stimulation. Thus, over the whole recording period, hundreds of data files were recorded. Information specific for each session (e.g. weekday, the chosen task condition, mood of the monkey) was first registered into a handwritten notebook and later transferred to the recording specific spreadsheets (label 7 in Figure 1, and Table 2).

1.4 The recording procedure and main preprocessing steps

The experimental setup (illustrated in Figure 1) was composed of two streams of signals: A) the recording and processing of neuronal signals (yellow arrows), B) the task control and recording of behavioral events (green and blue arrows).

The flow of the neuronal signals (stream A, yellow) started with cortical recordings with the Utah array. The signals from each active electrode were transmitted to a high density connector fixed to the skull. They were then processed by a headstage, attached directly to the connector, to improve the signal-to-noise ratio. The type of headstage was specified in the recording specific spreadsheet (label 7 in Figure 1 and Table 2) after each corresponding recording day. The signals were then transferred to the Front-End Amplifier to be amplified (gain factor: 5000), hardware-filtered (band-pass with cutoff frequencies 0.3 Hz and 7.5 kHz) and digitized (30 kHz). The hardware information about the Front-End Amplifier was entered into the Blackrock configuration spreadsheet (label 4 in Figure 1, and Table 2) at the beginning of the project. These processed 'raw' signals were transmitted to the Neural Signal Processor (NSP) via an optic fiber. The NSP was controlled by Central Suite (data acquisition software of Blackrock Microsystems) running under Windows on the data acquisition PC. Within the NSP the signals were further processed and saved to disk

into two output streams: (i) a direct output stream which was saved as .ns6 file (monkey N) or .ns5 (monkey L and T) depending on the version of Central Suite (for both see label 6a in Figure 1 and Table 2), and (ii) a downsampled (1 kHz) and digitally low-pass filtered (cutoff frequency 250 Hz) output stream designed to capture the LFP which was saved as .ns2 file (label 6b in Figure 1, and Table 2). Information about how the signals were processed and saved was distributed over several source files. Before the recording period of each monkey, the hardware properties of the NSP and general information about Central Suite and the data acquisition PC were entered into the Blackrock configuration spreadsheet (label 4). The hardware settings of the NSP defined by Central Suite were, however, saved in the recording specific spreadsheet (label 7 in Figure 1 and Table 2) and in the data files (label 5, 6a, and 6b Figure 1 and Table 2).

In parallel to the continuously sampled neuronal signals, a high-resolution (30kHz) high-pass filtered signal stream (at 500Hz in monkey T and L, and 250Hz in monkey N) was used to identify and save spiking activities online. For this, a user-defined threshold on each recording channel was set via the spike sorting module of Central Suite for each session to extract potential spike shapes (waveforms). However, these thresholds were not modified during a session. The waveforms were saved in the .nev file (label 5 in Figure 1 and Table 2), together with their respective time stamps. The size of the extracted time window for the waveforms (1.6 ms in monkey T and L, and 1.3 ms in monkey N) was set for the complete recording period of each monkey and therefore saved in the Blackrock configuration spreadsheet (label 4 in Figure 1 and Table 2).

The actual sorting of the extracted waveforms into single unit (SUA) or multi unit activities (MUA) was performed as a semi-automatic preprocessing step via the Plexon offline Spike Sorter (Plexon Inc, Dallas, Texas, USA; version 3.3). The sorting results were saved in an additional .nev file by Plexon (label 8 in Figure 1 and Table 2). The assignment of unit IDs to noise, SUA and MUA was defined in a hand written spike sorting specific text file (label 9 in Figure 1 and Table 2). To assess the quality of the identified units, a characterization of their waveforms (e.g., amplitude, width, signal-to-noise ratio) was performed using a custom MATLAB program that stored the results in a .mat file (label 10 in Figure 1 and Table 2).

The behavioral signals (stream B, green and blue) were monitored and controlled in real-time by LabVIEW (software of the National Instruments Corporation, Austin, Texas, USA) which ran on a second PC. In parallel, the behavioral events (digitized by an Analog-to-Digital Converter of National Instruments, where required) and signals were fed into the NSP and saved along with the neuronal events (.nev file, label 5 in Figure 1 and Table 2) or analog signals (.ns2 file, label 6b in Figure 1 and Table 2). The behavioral analog signals, registered at the force and displacement sensors attached to the object, were later offline processed via a custom MATLAB program to extract the performed pulling force and the event times (OT, HS, and OR). The results and parameters used for this preprocessing step were saved in two .mat files (labels 11 and 12 in Figure 1 and Table 2).

Another standard preprocessing step was the quality control of the LFP signals by custom Python program (see Computer - Quality Check, Figure 1) for the elimination of individual trials (on all electrodes) or individual electrodes (in all trials) which were corrupted by large artifacts or noise. This procedure was semi-automatic, i.e. the experimenter needed to visually control and, if necessary, adjust the criteria (e.g., based on the variance of the LFP) and redo the analysis. The results and parameters of this preprocessing step were documented in a .hdf5 file (label 13 in Figure 1 and Table 2).

1.5 Summary of metadata sources

To transform these various metadata sources into a comprehensive metadata collection it is necessary to first reorganize them according to the following types of source files:

- one source file per experiment containing metadata which are valid for the whole experiment independent of the used subjects (in our example experiment this matches the project specific info spreadsheet, label 0 in Figure 1 and Table 2)
- at least one source file for each subject containing metadata which are subject specific (source files with label 1 in Figure 1 and Table 2 in our example experiment)
- at least one source file for each recording device containing metadata which are valid for the recording period with the corresponding device (source files with label 2 - 4 in Figure 1 and Table 2 in our example experiment)
- at least one source file per session containing recording specific metadata (source files with label 5 - 7 in Figure 1 and Table 2 in our example experiment)
- at least one source file for each preprocessing step of each recording containing metadata which are valid for a specific preprocessing of a specific recording (source files with label 8 - 13 in Figure 1 and Table 2 in our example experiment)

2 USING AN ODML METADATA COLLECTION

In the main text we described five use cases and showed along those the advantages of a standardized organization of metadata. Additionally, we provided guidelines for creating a comprehensive metadata collection. Here we now complement both sections with practical demonstrations. Note that the code presented can be written in a more compact way, but for better readability we provide a longer, more explicit code format.

2.1 Manual inspection

As described in use case 2 it can be quite useful to be able to manually inspect a metadata collection to get familiar with an experimental study. There are three ways of manually screening an odML file.

The first possibility to open an odML file would be to use a simple text editor (see Listing S-1). This is possible, because odML is based on XML which is a textual data format readable and editable with any available text editor. It is therefore a quick way to manually inspect or edit the content of an odML file, but the XML based representation is not convenient for large odML files.

A second possibility to view, but not edit an odML file is to open it via a web browser (Figure S-1). For this, one has to add the XML-schema file (odML.xsl) to the directory where the odML files are located before opening them to view. The XML-schema file is available for download on the odML website (termed 'metdataStylesheet' on http://www.g-node.org/projects/odml/tools). The schema translates the XML based representation of odML into HTML code which is then interpreted by the web browser into an interactive web page representation. The web page will show the tree structure of the Sections as a static table of contents at the top and below all Sections and their Properties as a flat content list. Each Section in the tree representation is a link to its corresponding flat content representation. This approach is very useful for screening and browsing through an odML file, especially if it is large and complex.

The third possibility to manually inspect or edit an odML file is to use the odML Editor (Figure S-2). The editor ('`odml-gui`') is part of the Python odML library. Here, the representation of the tree structure of the Sections is separated from the flat representation of its Properties. The editor window is subdivided into three parts. The Sections pane (upper left) displays a tree view of all Sections starting from the top level of the document, the Properties pane (upper right) displays a table containing the Name, Value and the Value

attributes of each Property (row) belonging to a selected Section in the Sections pane, and the attributes pane (bottom) displays the attributes of the current selected Section, Property or Document. The header of the attributes pane indicates the path to the selected Section or Property in red starting from the Document root.

2.2 Navigating the odML structure

Depending on the experiment, the odML structure can become large and complex, thus making it difficult to find certain metadata within this complex structure. For this reason the odML Python library provides helper functions which can be used to find and extract metadata values with minimal user knowledge on the odML structure. In the following we will demonstrate how these helper functions, `itervalues()`, `iterproperties()` and `itersections()` (collectively referred to as iter functions), can be used in the scenario we defined for the use cases. For these demonstrations, we assume that an odML metadata collection for the reach-to-grasp study was already generated resulting in one odML file per session.

Bob wrote an analysis script to test if the firing rates depend on the behavioral condition in the trials, and he wants to run his analysis script on single unit (SUA) data pooled across sessions. Thus, he needs to check which recording sessions were already spike sorted. From a previous manual inspection of the odML files, he remembers that the Property containing this information was called “IsSpikeSorted”. He also remembers that this Property name is unique and that the type of the metadata Value saved in this Property is a boolean (True or False). He cannot remember where this Property is located in the complete tree structure of the odML files (for an example on how to extract odML objects via their absolute path in the hierarchy, see the odML Python tutorial at http://g-node.github.io/python-odml/). His knowledge is sufficient enough, though, to make use of the Python odML helper function `iterproperties()` which iterates through all Property objects of an odML file and combines it with a filter function that checks for each Property object if its name is equal to “IsSpikeSorted” (Listing S-2). He knows that this will give him a list containing exactly one Property containing the requested metadata. He extracts the Property from the list and accesses the single Value object of the Property to extract the stored metadata of type boolean to print out if the session he looked at was spike sorted or not.

If an odML Property or Section name is ambiguous, one can extend the filter function to check for several attributes of the requested object. For example, Bob wants to know the SUA IDs of one particular electrode with the ID 11. Again from previous inspections of the odML file, he remembers that the Property name which contains the metadata he is searching for is “SUAIDs” and that it exists as a child object below each of 96 uniquely named Sections which represent the active electrodes of the Utah array (cf., Figure 6). He makes use of this fact and extends the filter function not only to make sure that the property name is “SUAIDs”, but also that the name of the section of his particular electrode “Electrode_011” occurs in the path of the requested property (see Listing S-3). Bob combines this more complex filter function with the odML helper function `iterproperties()` and extracts the requested Property from the resulting list. He is aware that the Property “SUAIDs” can contain multiple Values which he writes into a list. He then loops through this list to access the Values containing the SUA IDs of Electrode_011.

Which iter function one has to use, and how complex the filter function should be, depends on both the structure of the odML file and the user’s need. For automatic extraction of metadata one has to make sure that the filter function is complex enough to guarantee that the iter function returns only the requested objects. In case of a large odML structure one should narrow the search down to a certain branch of the odML file and avoid iterations through Value objects of the odML. Both will save run time in the implementation.

The search for the Property "SUAIDs" of Section "Electrode_011", for example, could have been also written differently, as shown in Listing S-4. Bob knows that, although the Property name "SUAIDs" exists many times within the odML file, the Section name "Electrode_011" is unique and below this Section only one Property is named "SUAIDs". He can make use of this fact by dividing the search for the requested SUA IDs in two steps. In step one, Bob creates a filter function in combination with the odML helper function `itersections()` to find and extract the Section with name "Electrode_011" from the odML file. In the second step, he uses the odML helper function `iterproperties()` not on the entire odML file (`odml_of_recordingXX`), but on the extracted Section "Electrode_011" in combination with a filter function for finding the Property "SUAIDs".

Bob can also make use of the ambiguity of the odML Property name "SUAIDs" to collect the number of SUA IDs identified for all electrodes of the Utah array, which gives an idea about the quality of the spike detection and the spike sorting (see Listing S-5). Therefore he would combine the odML helper function `iterproperties()` with a filter function that only checks if the odML Property name equals "SUAIDs" and counts, based on the resulting lists of odML Properties with name "SUAIDs", how many odML Values contain metadata matching SUA IDs.

2.3 Navigating across odML files

In the previous subsection we demonstrated how to locate and access specific metadata in a given odML hierarchy. Here, we will illustrate how to apply this mechanism across odML files.

Let us assume that Bob defined his iter and filter function to find out whether a given recording session is spike sorted (see Listing S-6). He also extracted a list of all odML file names and knows that they match the names of the corresponding data files. He loops over all odML files and extracts for each if the session was spike sorted. If so, he appends the corresponding filename automatically to the list of spike sorted sessions.

If Bob has more than one criterion to be used for selecting sessions or even data from sessions, such as in use case 3, it is helpful to combine all checks based on the `iterproperties()` function in a single criterion function for increased clarity. Based on the code examples in Listing S-2 and Listing S-5, Bob may create a criterion function which checks if a session was spike sorted and if so, if the number of identified single units was larger than 60 (Listing S-7).

2.4 Integration of additional metadata

Additional preprocessing steps (e.g. spike sorting or quality assessment) as described in Section 1 and use case 1 (Section 3.1 of the main text) are often performed over a time period of months after the actual recording which is typical for a workflow of an electrophysiological experiment. Along use case 1 we will now illustrate different scenarios of how metadata of such preprocessing procedures can be gradually integrated into an existing odML file.

In a first scenario, the preprocessing step is expected and known in advance (e.g. spike sorting). Here, the odML structure can be planned ahead with default dummy metadata Values in the form of an odML template. In this case it is possible to replace the dummy Values in the odML structure by the upcoming actual metadata Values of the preprocessing step.

Alternatively, the preprocessing step may not be expected, for example, if its importance arises only after performing preliminary analyses of the data. Indeed, the ideal odML structure for metadata may only become clear during development of a new preprocessing step and needs to be integrated into existing odML files later on. In such a case one should update the original odML template structure with the

new preprocessing structure and rerun the generation of all odML files to keep overall consistency and reproducibility.

2.5 odML access via MATLAB

In use case 5 we discussed a situation where two scientists (Alice and Carol) from different labs decide to work together even though they use different programming languages for data analysis (MATLAB and Python, respectively). The question arises how both scientists can use odML without abandoning their preferred programming language. Indeed, odML libraries exist not only for Python but also for MATLAB and Java. As MATLAB is often used in experimental neurophysiological laboratories, we illustrate here how the previously stated Python code examples can be translated into MATLAB.

The current version of the MATLAB odML library provides an API that differs from the python-odml library. In particular the MATLAB API is limited to load data from odML files but not to write to odML files, and the flexible `iterproperties()` is replaced by a comparable, but more limited, helper function called `odml_find()`.

When using the MATLAB odML library (https://github.com/G-Node/matlab-odml), the odML data are stored in MATLAB structure arrays, making handling of the data convenient and familiar for MATLAB users. It must be noted that the `odml_load()` loading function provides an option to choose between two possible ways of mapping the odML data to the fields of the structure array. Using the default 'tree' option, the fields of the structure array are directly named after the names of the Sections and Properties defined in the odML file, as illustrated in Listing S-8. Using the 'odml' option, the odML data are loaded in the structure array following more closely the odML object model, as illustrated in Listing S-9.

As it can be inferred by comparing the code in Listing S-8 and Listing S-9, using one option or the other is more suitable depending of the type of processing that will be performed on the metadata. For example, when the user knows the odML hierarchy and wants to access directly a given Property, the option 'tree' leads to more explicit naming in the structure array fields which makes writing and reading of the code more convenient (compare `rootSection.Subject.Weight.value` and `rootSection.section(1).property(2).value(1).value` from Listing S-8 and Listing S-9, respectively). For some more advanced processing, in particular when looping over metadata structures, or when the number of Values or Properties or Sections is unknown, the 'odml' option can be more suitable. A more detailed discussion about the two loading options can be found in the help of the `odml_load()` function.

The `odml_find()` function searches an odML tree for the Section or Property object with a given name (`object = odml_find(odml_tree, object_name)`) or all sections and property objects with the given name (`object_list = odml_find(odml_tree, object_name, Inf)`). It can be used to build the MATLAB code equivalent to the Python code that we gave in our previous examples.

In the simple case where the requested object is uniquely represented in the odML file, as it is the case in our first code example in which Bob wants to find out if a particular recording session was spike sorted (Listing S-2), the MATLAB code is straight-forward (Listing S-10).

In the more complex situation where the requested object is not uniquely represented in the odML file, as in our second code example in which Bob wants to know the SUA IDs of the electrode with ID 11, we need to extract first the unique identifiable electrode Section "Electrode_011" and then use the resulting Section object in the `odml_find()` function to access the metadata of Property "SUAIDs" (Listing S-11). This approach is not as flexible as the Python code using the combination of `iterproperties()` with

complex filter functions as demonstrated in Listing S-3, but it is very similar to the approach in Listing S-4 and still powerful enough to access any metadata within the odML file with little detail knowledge on the odML file structure.

Finally, as illustrated in listing Listing S-12, we can also make use of the `odml_find()` function to find a list of Properties with ambiguous names. For example, we may wish to collect the number of SUAIDs identified for all electrodes of the Utah array, as we did in the code example in Listing S-5.

REFERENCES

Riehle, A., Wirtssohn, S., Grün, S., and Brochier, T. (2013). Mapping the spatio-temporal structure of motor cortical LFP and spiking activities during reach-to-grasp movements. *Front Neural Circuits* 7, 48. doi:10.3389/fncir.2013.00048

3 SUPPLEMENTARY TABLES AND FIGURES

odML - Metadata

Document info

Author: Bob
Date:
Version:
Repository:

Structure

- Subject (type: subject)
  - ArrayImplant (type: subject/preparation)

Content

Section: Subject

Type: subject
Id:
Repository:
Link:
Include:
Definition: Information on the investigated experimental subject (animal or person)
Mapping:

Name	Value	Uncertainty	Unit	value id	Type	Comment	Definition
Species	Macaca mullata				string		Binomial species name (genus, species within genus)

top

Section: ArrayImplant

Type: subject/preparation
Id:
Repository:
Link:
Include:
Definition: Information on the array implant performed on subject
Mapping:

Name	Value	Uncertainty	Unit	value id	Type	Comment	Definition
Date	2011-09-30				date		Date of the surgery

top

Figure S-1. HTML view of an odML file. The displayed odML document Subject_Demo.odml is schematically displayed in Figure 5, its corresponding Python implementation is shown in Figure 8, and the XML-based representation is demonstrated in Listing S-1.

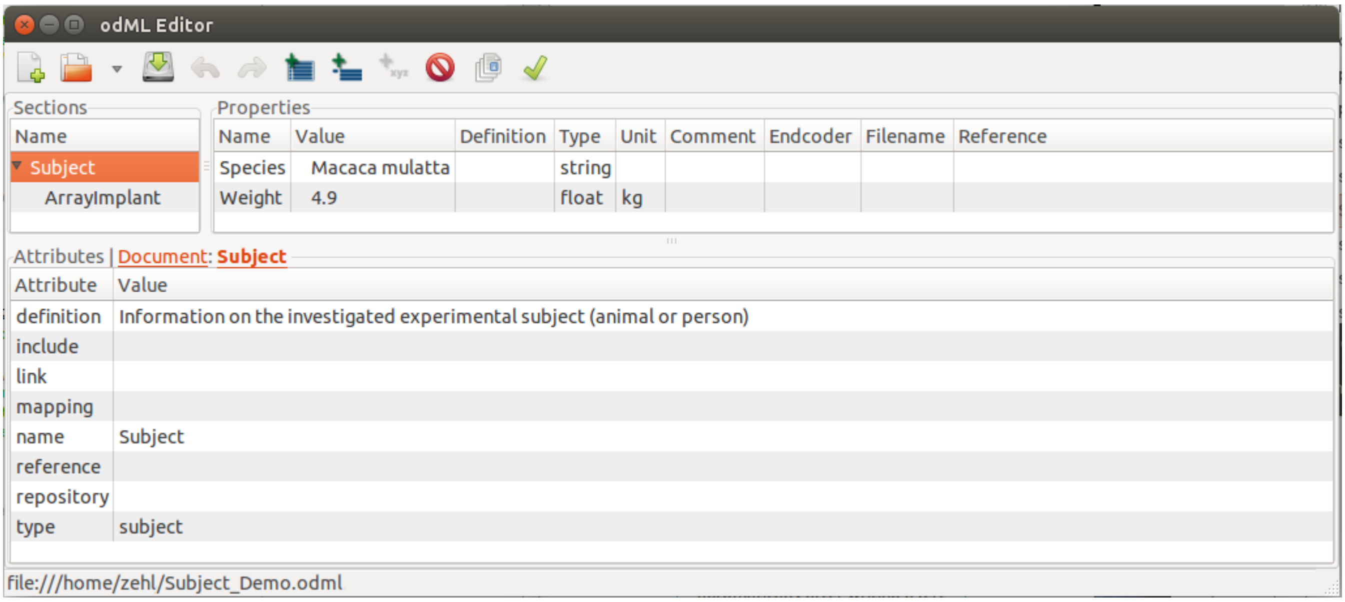

Figure S-2. odML Editor view of an odML file. The displayed odML document Subject_Demo.odml is schematically displayed in Figure 5, its corresponding Python implementation is shown in Figure 8, and the XML-based representation is demonstrated in Listing S-1. Note that the 'Subject' section was selected (marked in orange in the sections pane). The corresponding properties of the selected section ('Species' and 'Weight') are displayed in the properties pane.

```xml
<?xml version="1.0" encoding="UTF-8"?>
<?xml-stylesheet type="text/xsl" href="odmlTerms.xsl"?>
<?xml-stylesheet type="text/xsl" href="odml.xsl"?>
<odML version="1">
  <section>
    <definition>Information on the investigated experimental subject (animal or person)</definition>
      <property>
        <definition>Binomial species name (genus, species within genus)</definition>
        <value>Macaca mulatta<type>string</type></value>
        <name>Species</name>
      </property>
      <property>
        <definition>Body weight of the subject.</definition>
        <value>4.9<unit>kg</unit><type>float</type></value>
        <name>Weight</name>
      </property>
    <name>Subject</name>
    <section>
      <definition>Information on the array implant performed on subject</definition>
      <property>
        <definition>Date of the surgery</definition>
        <value>2011-09-30<type>date</type></value>
        <name>Date</name>
      </property>
      <name>ArrayImplant</name>
      <type>subject/preparation</type>
    </section>
    <type>subject</type>
  </section>
  <author>Bob</author>
</odML>
```

Listing S-1. XML-based representation of an odML file. XML-based representation of the odML Document Subject_Demo.odml which is schematically displayed in Figure 5. The corresponding Python code is shown in Figure 8.

```python
import odml

# load an odML file of one recording
odml_filename = '1101126-002.odml'
odml_of_recording = odml.tools.xmlparser.load(odml_filename)

# define a filter function which checks if the name of object (x) equals
# "IsSpikeSorted"
def filter_function(x):
    return x.name=="IsSpikeSorted"

# combine iter and filter function to collect all Properties fullfilling the
# given condition
list_of_properties = odml_of_recording.iterproperties(filter_function)

# if you know the Property is unique, extract it from this list
wanted_property = list_of_properties[0]

# if you know it contains only a single Value, extract it from the wanted
# Property
value_of_wanted_property = wanted_property.value

# extract the metadata (here, boolean) from the Value
metadata = value_of_wanted_property.data


# remove extension from odml_filename
odml_filename_rmext = os.path.splitext(odml_filename)[0]
# get only filename of recording
recording_filename = os.path.basename(odml_filename_rmext)

# print out if recording was spike sorted
if metadata == True:
    print("recording %s was spike sorted" % recording_filename)
else:
    print("recording %s is NOT yet spike sorted" % recording_filename)
```

Listing S-2. Extract unique objects from an odML file in Python. Python code for extracting an odML object with a unique name. The example demonstrates how one makes use of a filter function for the object name ("IsSpikeSorted") in combination with the object corresponding Python odML function `iterproperties()`.

```python
import odml

# load an odML file of one recording
odml_filename = '1101126-002.odml'
odml_of_recording = odml.tools.xmlparser.load(odml_filename)

# define a filter function which checks if the name of object (x) equals
# "SUAIDs" and the Section name "Electrode_011" occurs in the object's path
def filter_function(x):
    return x.name == "SUAIDs" and "Electrode_011" in x.get_path()

# combine iter and filter function to collect all Properties fullfilling the
# given condition
list_of_properties = odml_of_recording.iterproperties(filter_function)

# if you know the Property is unique, extract it from this list
wanted_property = list_of_properties[0]

# if you know it contains multiple Values, extract them as list from the
# wanted Property
list_of_values_of_wanted_property = wanted_property.values

# loop through the list of Values to access metadata and print out SUA IDs of
# Electrode_011
print("SUA IDs of Electrode_011:")
for value_of_wanted_property in list_of_values_of_wanted_property:
    metadata = value_of_wanted_property.data
    print(metadata)
```

Listing S-3. Extract ambiguous objects from an odML file in Python via search conditions. Python code for extracting an odML object with an ambiguous name by extending the conditions given in the filter function.

```python
import odml

# load an odML file of one recording
odml_filename = '1101126-002.odml'
odml_of_recording = odml.tools.xmlparser.load(odml_filename)

# define a filter function which checks if the name of object (x) equals
# "Electrode_011"
def filter_function_sec(x):
    return x.name == "Electrode_011"

# combine iter and filter function to collect all Sections fullfilling the
# given condition
list_of_sections = odml_of_recording.itersections(filter_function_sec)

# if you know the Section is unique, extract it from this list
wanted_section = list_of_sections[0]

# define a new filter function which checks if the name of object (x) equals
# "SUAIDs"
def filter_function_prop(x):
    return x.name == "SUAIDs"

# use iter function on the wanted Section only to filter all Properties
# fullfilling the given condition
list_of_properties = wanted_section.iterproperties(filter_function_prop)

# if you know the Property is now unique, extract it from this list
wanted_property = list_of_properties[0]

# if you know it contains multiple Values, extract them as list from the
# wanted Property
list_of_values_of_wanted_property = wanted_property.values

# loop through the list of Values to access metadata and print out SUA IDs of
# Electrode_011
print("SUA IDs of Electrode_011:")
for value_of_wanted_property in list_of_values_of_wanted_property:
    metadata = value_of_wanted_property.data
    print(metadata)
```

Listing S-4. Extract ambiguous objects from an odML file in Python via partial searches. Python code for extracting an odML object with an ambiguous name by narrowing down the search to a smaller part of the odML document.

```python
import odml

# load an odML file of one recording
odml_filename = '1101126-002.odml'
odml_of_recording = odml.tools.xmlparser.load(odml_filename)

# define a filter function which checks if the name of object (x) equals
# "SUAIDs"
def filter_function(x):
    return x.name == "SUAIDs"

# combine iter and filter function to collect all Properties fullfilling the
# given condition
list_of_properties = odml_of_recording.iterproperties(filter_function)

# make use of the list of all Properties named 'SUAIDs' to identify how many
# single units were identified on all electrodes
suaid_number = 0
for prop in list_of_properties:
    list_of_values = prop.values
    for val in list_of_values:
        metadata = val.data
        if metadata in range(1, 17):
            suaid_number += 1

# print how many SUA IDs were identified in this recording
print("Number of SUA IDs:", suaid_number)
```

Listing S-5. Extract all ambiguous objects from an odML file in Python. Python code for extracting a list of related odML objects with ambiguous names.

```python
import os
import odml

# define the directory to the odml files of all recordings
directory_of_odmlfiles = os.getcwd()

# get all odml filenames
list_odml_filenames = os.listdir(directory_of_odmlfiles)

# create an empty list for filenames of spike sorted recordings
list_spikesorted_filenames = []

# define a filter function which checks if the name of object (x) equals
# "IsSpikeSorted"
def filter_function(x):
    return x.name == "IsSpikeSorted"

# loop through the extracted list of odml filenames
for odml_filename in list_odml_filenames:
    # load odml file of recording
    odml_of_recording = odml.tools.xmlparser.load(odml_filename)

    # combine iter and filter function to collect all Properties
    # fullfilling the given condition
    list_of_properties = odml_of_recording.iterproperties(filter_function)

    # if you know the Property is unique, extract it from this list
    wanted_property = list_of_properties[0]

    # if you know it contains only a single Value, extract it from the
    # wanted Property
    value_of_wanted_property = wanted_property.value

    # extract the metadata (here, boolean) from the Value
    metadata = value_of_wanted_property.data

    # append to list if recording was spike sorted
    if metadata == True:
        # remove extension from odml_filename
        odml_filename_rmext = os.path.splitext(odml_filename)[0]
        # get only filename of recording
        recording_filename = os.path.basename(odml_filename_rmext)

        # append recording_filename to list of spike sorted recordings
        list_spikesorted_filenames.append(recording_filename)
```

Listing S-6. Extract a specific object from an odML file in Python used in a data selection. Python code to generate a list of filenames of spike sorted recording sessions.

```
import os
import odml

# define a filter function which checks if the name of object (x) equals
# "IsSpikeSorted"
def is_spikesorted(x):
    return x.name == "IsSpikeSorted"

# define a filter function which checks if the name of object (x) equals
# "SUAIDs"
def name_is_suaids(x):
    return x.name == "SUAIDs"

# define criteria function
def criteria_function(odml_of_recording):
    """Checks if a recording was spike sorted, and if yes, if the number
    of identified single units is larger than 60"""

    # combine iter and filter function to collect all Properties fullfilling
    # the given condition
    list_of_properties = odml_of_recording.iterproperties(is_spikesorted)

    # if you know the Property is unique, extract it from this list
    wanted_property = list_of_properties[0]

    # if you know it contains only a single Value, extract it from the wanted
    # Property
    value_of_wanted_property = wanted_property.value

    # extract the metadata (here, boolean) from the Value
    metadata = value_of_wanted_property.data

    if metadata == True:
        # combine iter and filter function to collect all Properties
        # fullfilling the given condition
        list_of_properties = odml_of_recording.iterproperties(name_is_suaids)

        # make use of the list of all Properties named 'SUAIDs' to identify
        # how many single units were identified on all electrodes
        suaid_number = 0
        for prop in list_of_properties:
            list_of_values = prop.values
            for val in list_of_values:
                metadata = val.data
                if metadata in range(1, 17):
                    suaid_number += 1
        if suaid_number > 60:
            return True
        else:
            return False
    else:
        return False

# define the directory to the odml files of all recordings
directory_of_odmlfiles = os.getcwd()

# get all odml filenames
list_odml_filenames = os.listdir(directory_of_odmlfiles)

# create an empty list for filenames of spike sorted recordings with a
# sufficient number of single units
list_filenames = []

# loop through the extracted list of odml filenames
for odml_filename in list_odml_filenames:
    # load odml file of recording
    odml_of_recording = odml.tools.xmlparser.load(odml_filename)

    # use the defined criteria function to test if recording was spike sorted
    if criteria_function(odml_of_recording) == True:
        # remove extension from odml_filename
        odml_filename_rmext = os.path.splitext(odml_filename)[0]
        # get only filename of recording
        recording_filename = os.path.basename(odml_filename_rmext)

        # append recording_filename to list of spike sorted recordings
        list_filenames.append(recording_filename)
```

Listing S-7. Extract multiple objects from an odML file in Python used in a data selection. Python code that uses a criterion function to generate a list of filenames of spike sorted recording sessions which contain at least 60 identified units.

```
odml_config;

% load the test odML file with the default 'tree' option
rootSection = odml_load('example_odML.odml');

% access the value of the weight of the subject
weight = rootSection.Subject.Weight.value;
```

Listing S-8. odML in MATLAB - 'tree' option. MATLAB code for accessing a specific metadata value when using the default 'tree' loading option. The content of the file 'listing2.odml' is given in Figure 5.

```
odml_config;

% load the test odML file with the 'odml' option
rootSection = odml_load('example_odML.odml', 'odml');

% access the value of the weight of the subject
weight = rootSection.section(1).property(2).value(1).value;
```

Listing S-9. odML in MATLAB - 'odml' option. MATLAB code for accessing a specific metadata value when using the 'odml' loading option. The content of the file 'listing2.odml' is given in Figure 5.

```
odml_config;

% load an odML file of one recording
odml_filename = '1101126-002.odml';
odml_of_recording = odml_load(odml_filename);

% if you know that the Property name 'IsSpikeSorted' is unique, extract the
% corresponding Property
wanted_property = odml_find(odml_of_recording, 'IsSpikeSorted');

% extract the metadata (here, boolean) from the Property, knowing it
% contains a single value
metadata = wanted_property.value;

% printout if recording was spike sorted
if metadata == true
    disp('recording was spike sorted');
else
    disp('recording is NOT yet spike sorted');
```

Listing S-10. Extract unique objects from an odML file in MATLAB. MATLAB code for extracting an odML object with a unique name.

```
odml_config;

% load an odML file of one recording
odml_filename = '1101126-002.odml';
odml_of_recording = odml_load(odml_filename, 'odml');

% if you know that the Section name 'Electrode_011' is unique, extract the
% corresponding Section
wanted_section = odml_find(odml_of_recording, 'Electrode_011');

% if you know that the Property name 'SUAIDs' is unique in the wanted
% Section, extract the corresponding Property
wanted_property = odml_find(wanted_section, 'SUAIDs');

% if you know it contains multiple Values, extract them as struct from the
% wanted Property
struct_of_values_of_wanted_property = wanted_property.value;

% loop through the struct of Values to access metadata and printout SUA IDs
% of Electrode_011
disp('SUA IDs of Electrode_011:');
for ind = 1:length(struct_of_values_of_wanted_property)
    value_of_wanted_property = struct_of_values_of_wanted_property(ind);
    metadata = value_of_wanted_property.value;
    disp(metadata);
end
```

Listing S-11. Extract ambiguous objects from an odML file in MATLAB. MATLAB code for extracting an odML object with an ambiguous name.

```
odml_config;

% load an odML file of one recording
odml_filename = '1101126-002.odml';
odml_of_recording = odml_load(odml_filename, 'odml');

% extract all the Properties having the name 'SUAIDs'
list_of_properties = odml_find(odml_of_recording, 'SUAIDs', Inf);

% make use of the list of all Properties named 'SUAIDs' to identify how
% many single units were identified on all electrodes
suaid_number = 0;
for ind_prop = 1 :length(list_of_properties)
    prop = list_of_properties{ind_prop};
    struct_of_values = prop.value;
    for ind_val = 1:length(struct_of_values)
        val = struct_of_values(ind_val);
        metadata = val.value;
        if metadata >= 1 && metadata <= 17
            suaid_number = suaid_number + 1;
        end
    end
end

% print how many SUAIDs were identified in this recording
disp('Number of SUAIDs:');
disp(suaid_number);
```

Listing S-12. Extract all ambiguous objects from an odML file in MATLAB. MATLAB code for extracting a list of related odML objects with ambiguous names.
